# Enhanced measurement of residual chemical shift anisotropy for small molecule structure elucidation†

**DOI:** 10.1039/c8cc00552d

**Published:** 2018-04-24

**Authors:** Yizhou Liu, Ryan D. Cohen, Kirk R. Gustafson, Gary E. Martin, R. Thomas Williamson

**Affiliations:** a Structure Elucidation Group, Process and Analytical Research and Development, Merck. & Co. Inc., 126 East Lincoln Avenue, Rahway, NJ 07065, USA.; b Molecular Targets Program, Center for Cancer Research, National Cancer Institute, Frederick, Maryland 21702-1201, USA

## Abstract

A method is introduced to measure residual chemical shift anisotropies conveniently and accurately in the mesophase of poly-γ-(benzyl-_L_-glutamate). The alignment amplitude is substantially enhanced over common methods which greatly benefits measurements particularly on sp^3^ carbons. The approach offers significant improvements in data accuracy and utility for small molecule structure determination.

Residual chemical shift anisotropy (RCSA) arises from incomplete rotational averaging of chemical shift anisotropy (CSA) as a molecule undergoes partial ordering in an alignment medium.^1–5^ RCSA provides structural information in the form of relative orientations between different CSA tensors in a molecule. Such information is particularly valuable for proton-deficient compounds in which one-bond ^13^C–^1^H residual dipolar couplings (RDCs) are scarce. Historically, accurate RCSA measurements were plagued by the change in the isotropic component of the chemical shift tensor (ΔΔ*δ*_iso_) as a molecule is aligned by the addition of an alignment medium. Strategies have recently been introduced to eliminate or correct for ΔΔ*δ*_iso_ for RCSA data collected in polymeric gels.^6–8^ While RCSAs from these methods, either alone or in conjunction with RDC data, have been successfully applied to the determination of constitution and configuration of a number of small molecules,^7–11^ a limitation exists in that RCSA values tend to be very small for sp^3^-hybridized carbons under these alignment conditions, and differentiation of subtle structural variations involving these carbons is expected to be challenging in the absence of RDC data.

Increasing the alignment amplitude is a logical step to address the problem of small RCSAs. In fact, certain liquid crystal (LC) media, such as that formed by poly-γ-(benzyl-_L_-glutamate) (PBLG),^12^ are known to generate alignment amplitudes over an order of magnitude larger than constrained polymeric gels.^13^ For RDC measurement, a stronger alignment is not necessarily beneficial, as it simultaneously enlarges undesired interactions such as the homonuclear dipolar couplings, which severely broaden proton lines and degrade the performance of HSQC-type experiments typically used for RDC measurements. As a result, PBLG has only occasionally been used as an alignment medium for acquiring RDC data in structure elucidation studies.^14–19^ In contrast, RCSA measurement only utilizes the simple ^13^C{^1^H} experiment in which inhomogeneous ^13^C–^1^H dipolar interactions and homogenous interactions from proton spin diffusion are both eliminated by proton decoupling. Therefore, relatively sharp ^13^C lines can still be obtained even with strong alignment, making accurate RCSA measurement feasible. In fact, the RCSA phenomenon has previously been utilized in the chiral nematic (cholesteric) phase of PBLG for the qualitative differentiation of enantiomeric mixtures.^20–23^ Here we describe a simple but highly effective strategy for the accurate quantitative measurement of RCSA in PBLG mesophase for the purpose of small molecule structural elucidation. Data were collected for strychnine, retrorsine, and caulamidine A (Fig. 1a–c), for which RCSA data were also available for comparison from stretched poly-methyl-methacrylate (PMMA) or poly-2-hydroxyethyl-methacrylate (PHEMA) gels.^8,11^

The challenge for accurate RCSA measurement is to correct for ΔΔ*δ*_iso_ associated with the PBLG phase transition at different concentrations. Our correction method is based on the premise that the ΔΔ*δ*_iiso_ dependence on PBLG concentration is identical in both phases, such that the ΔΔ*δ*_iiso_ in the mesophase can be corrected for by extrapolating its value from its trend in the isotropic phase. The rationale and limitation of this approach are discussed in ESI.†

We successively added small amounts of PBLG to a strychnine solution and acquired a ^13^C{^1^H} spectrum after each addition (Fig. 2). Tetramethylsilane (TMS) at 4% (v/v) was added for ^13^C chemical shift referencing. The effect of the bulk susceptibility change at different PBLG concentrations is eliminated by TMS referencing. Although vibration-rotation coupling can generate anisotropic effects in a tetrahedral molecule,^24–27^ TMS aligns much more weakly than most compounds of interest and can therefore be used as a convenient reference in most cases.^8^ Alternatively, RCSA measurements can also be referenced by an arbitrary carbon in the compound itself, following a previously described procedure.^7^ As shown in Fig. 2a, the C21 sp^2^ resonance of strychnine gradually shifted upfield as PBLG was added up to 8.8% (w/v) due to ΔΔ*δ*_iso_, and no CDCl_3_ residual quadrupolar coupling (RQC) was observed consistent with an isotropic solution. As the PBLG concentration reached 11.4%, a sudden large downfield resonance jump occurred due to RCSA (spectrum 5) concomitant with a CDCl_3_ RQC of 215.5 Hz, indicating the sample entered the mesophase. Additional PBLG increased both RCSA and RQC as the alignment strengthened. As shown in Fig. S3 (ESI†), ΔΔ*δ*_iso_ follows a nearly linear relationship with PBLG concentration within the isotropic phase, although not strictly. The linearity is actually expected if the analyte and PBLG do not bind *(i.e.*, the binding *K*_D_ is very large). This relationship is then used to remove the ΔΔ*δ*_iso_ contribution in the mesophase. Importantly, because ΔΔ*δ*_iso_ from PBLG addition is small in comparison to RCSA even for sp^3^ carbons (Fig. 2b), ignoring the surface interference effect, which is a higher order effect as described in the ESI,† is not expected to cause large errors, at least when the PBLG concentration is not much higher than the mesophase critical concentration (*C*_crit_).

Based on the linear relationship, we propose a simplified three-measurement approach, using the terminology: I0–I1–A1. I0 is an isotropic measurement without PBLG, I1 is a second isotropic measurement at a PBLG concentration below but close to C_crit_, and A1 is an anisotropic measurement at a PBLG concentration above C_crit_. RCSA can be extracted from these measurements by the simple equation:

(1) $${\text{RCSA}}_{i}=\left(\delta_{\text{A}1}^{i}-\delta_{\text{A}1}^{\text{TMS}}\right)-\left(\delta_{\text{I}0}^{i}-\delta_{\text{I}0}^{\text{TMS}}\right)\;-\;\frac{{\left[\text{PBLG}\right]}_{\text{A}1}}{{\left[\text{PBLG}\right]}_{\text{I}1}}\left[\left(\delta_{\text{I}1}^{i}-\delta_{\text{I}1}^{\text{TMS}}\right)-\left(\delta_{\text{I0}}^{i}-\delta_{\text{I0}}^{\text{TMS}}\right)\right]$$

The first and second terms in parentheses on the right side of eqn (1) represent the TMS-referenced chemical shifts of carbon *i* in A1 and I0, respectively. Their difference defines the RCSA of carbon *i*, as well as ΔΔ*δ*_iso_, represented by the third term. Note that all chemical shift terms on the right are determined experimentally and the PBLG concentrations are also known. Therefore, the RCSA value of each carbon can be extracted.

As shown in Fig. 2c, the RCSA in PBLG is much larger than the corresponding RCSA in a stretched PMMA gel.^8^ The ~2-fold increase in line-width is amply compensated for by the over 34-fold enhancement of the measured RCSA value. Consequently, these large RCSAs greatly improve the quality of data and structural analysis, as demonstrated later. One noteworthy point is that the proton decoupling used for an isotropic sample may be insufficient to fully decouple the much larger couplings present in mesophase. For example, the alignment with 34.5% PBLG can generate ^13^C-^1^H RDCs of over 1 kHz so higher decoupling power and a decoupling sequence with wide broadband coverage, such as GARP or WURST, should be used for PBLG mesophase (see ESI† for experimental details).

The data quality is evaluated in Table 1. The generalized degree of order (GDO) was employed to measure the overall magnitude of molecular ordering.^28^ For a mostly rigid molecule, the GDO measures the alignment amplitude. Our previously reported RCSA data collected in PMMA gels were used for comparison. Clearly, even at relatively low PBLG concentrations, the GDOs are ~5–6 times larger than in PMMA gels; at the high end of this study, a GDO of 2% was obtained for strychnine, *i.e*., the anisotropic NMR interactions were measured in a liquid sample at 2% of the solid-state values. To the best of our knowledge, anisotropic interactions of this magnitude have not been previously demonstrated in high-resolution NMR for complex molecules.

RCSAs from these strong alignments yield significantly improved structural analysis, as reflected by the *Q*-factors. As shown in Table 1, *Q*-factors with all carbon atoms considered (*Q*_all_) are substantially better in PBLG mesophases than in PMMA gels. For example, a *Q*_all_ of 0.029 in 15.5% PBLG is a significant improvement over a *Q*_all_ of 0.049 in the stretched PMMA gel for strychnine. Similarly, RCSAs from the rigid part of retrorsine produced a vastly improved *Q*_all_ of 0.033 in 15.1% PBLG, over a *Q*_all_ of 0.104 in stretched PMMA.

This strong alignment is particularly beneficial for compounds constituted primarily of sp^3^ carbons. We demonstrate this point by omitting all RCSA data from sp^2^ carbons while using only those data from sp^3^ carbons to calculate the *Q*-factors (*Q*_sp_^3^) and alignment tensor parameters. As shown in Table 1, the improvement in *Q*_sp_^3^ followed the trend of *Q*_all_ for data in PBLG *vs*. PMMA gels, with *Q*_sp_^3^ from the former being lower than 0.1 in most cases. To further evaluate the reliability of using only sp^3^ carbon RCSAs for structural analysis, we compared the agreement of the alignment tensor parameters determined with all of the data and those determined with only sp^3^ data through calculations of their intertensor angles (*θ*).^29^ As shown in Table 1, the intertensor angles are substantially smaller in PBLG solutions than in PMMA gels, with *θ* well below 10° in most cases, confirming that the reliability of using only sp^3^ carbon data significantly improved. One exception was observed for retrorsine in 12.6% PBLG, in which y was slightly higher than in the PMMA gel, presumably due to inaccuracy of the alignment tensor determination with only a small number of sp^3^ carbons. From Table 1, it is also apparent that the *Q*_sp_^3^-factor under a larger alignment for strychnine at the high end of PBLG concentration did not improve, but instead slightly degraded, whereas the *Q*_all_-factor barely changes. This divergence between *Q*_all_ and *Q*_sp_^3^ is possibly due to smaller sp^3^ carbon RCSA values being more susceptible to imperfections in our ΔΔ*δ*_iso_ elimination method at high PBLG concentrations where surface interference is expected to be more pronounced. Based on this observation, for a sp^3^ carbon-rich compound, a PBLG concentration of about 15% is advisable based on available data.

In a recent study, we successfully differentiated 13 possible diastereomers of strychnine using only RCSA data collected in a PMMA gel.^8^ Here we conducted the same analysis with RCSAs collected in 15.5% PBLG as a representative example (Fig. 3a), and compared the performance of PBLG *vs*. PMMA gel data for this differentiation (Fig. 3b). The stereochemical definition follows that in our previous work. The correct diastereomer *RSSRRS* with a *Q*-factor of 0.029, shown on the left, is clearly distinguished from all other diastereomers that exhibited much larger *Q*-factors (Fig. 3a). The confidence level of differentiation can be quantitatively assessed by the ratio: *Q*/*Q*_best_, where *Q* is the *Q*-factor of an isomer being evaluated, and Q_best_ represents the lowest *Q*-factor in the pool,^30^ which, in this example, is *Q*_RSSRRS_ The larger this *Q*-factor ratio, the higher the level of confidence for the differentiation. Fig. 3b displays the *Q*-factor ratios of all 13 diastereomers over *Q*_RSSRRS_, for both PBLG data (blue) and PMMA gel data (red). Clearly, the confidence level is about twice as good with PBLG data for those less discriminated isomers, such as RSRRRS and SSSRSR, and overall much better, in comparison to the results obtained with PMMA gels. We should mention that the data quality from the stretched PMMA gel, with a *Q*_RSSRRS_ of 0.049, was actually quite good. However, that result is superseded by the enhanced quality afforded by the measurement in PBLG.

Next, as a more challenging example, we used RCSA data to differentiate caulamidine A (Fig. 1c) from its C26-inverted diastereomer. In a recent collaboration, we reported the structure of caulamidine A using NMR data including RDC and RCSA collected in a PHEMA gel.^11^ Although a combination of RDC and RCSA clearly favoured the proposed structure over other constitutional and stereoisomers, the weak alignment of caulamidine A in PHEMA clearly left room for further improvement. Indeed, alignment with 13.1% PBLG generated a large RCSA range of —40 to 100 Hz, a vast enhancement over the —3.4 to 3.5 Hz range in PHEMA. The second best structural candidate in the previous study was the C26-inverted diastereomer. This chiral inversion swaps the position of the chlorine atom but minimally perturbs the overall conformation (Fig. 4). Despite the high level of structural similarity, RCSA data from PBLG strongly supports the proposed structure with a *Q*-factor of 0.077 over the C26 inverted diastereomer with a *Q*-factor of 0.328 (Fig. 4). Interestingly, the largest RCSA outlier from the correlation plot of experiment *vs*. theory, (circled in red in Fig. 4b), corresponds to the inverted sp^3^ carbon 26, which clearly reveals the inconsistency of this chiral inversion with experimental data. The RCSA data were collected with only 1.6 mg of material, and the actual measurements took 54, 69, and 131 minutes for I0, I1, and A1, respectively, on a 600 MHz spectrometer with a 5 mm helium cryoprobe. This work further demonstrates the utility of the method for mass-limited samples.

Finally, anisotropic NMR work on scarce materials, such as many natural products, usually mandates sample recovery from the alignment medium after NMR measurements are completed. Here we also developed an efficient sample recovery procedure that achieves over 90% recovery for an array of test compounds soluble in ethyl acetate, as shown in Table S1 (ESI†). The recovery procedure is detailed in the ESI.†

In summary, we demonstrated with several examples that a simple first-order correction for PBLG concentration was sufficient to eliminate ΔΔ*δ*_iso_ from RCSA measurement in the PBLG mesophase. The large RCSAs resulting from the strong alignment greatly improve structural analysis in general, especially that of sp^3^ rich compounds. The commercial availability of PBLG, the facile sample preparation and high analyte recovery comparable to polymer gels, and the easily implemented NMR experiment should facilitate utilization of RCSA data for small molecule structural analysis in a greater number of laboratories than has been heretofore possible with existing methods.

## Supplementary Material

supplemental

## Figures and Tables

**Fig. 1:**
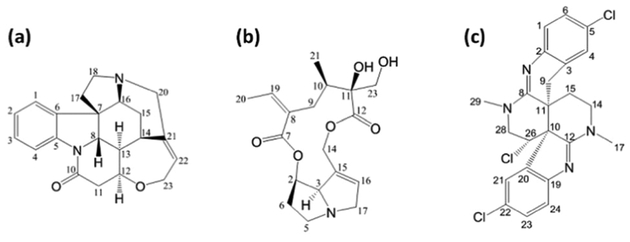
Structures of strychnine (**1a**), retrorsine (**1b**), and caulamidine A (**1c**).

**Fig. 2:**
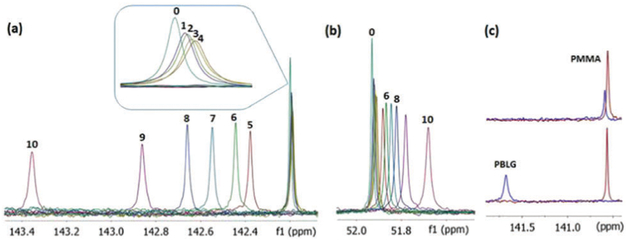
Representative spectra from RCSA measurement. Labelled spectra (0–10) were collected with 0, 2.1, 4.1, 6.6, 8.8, 11.4, 12.9, 15.5, 18.4, 22.9, and 34.5% (w/v) of PBLG in CDCl3, respectively. Samples 0–4 were acquired with PBLG below its LC-forming critical concentration and hence are isotropic solution spectra, whereas the data for samples 5–10 were collected with PBLG above its *C*_crit_ and are increasingly anisotropic in nature. A quaternary sp^2^ carbon of strychnine (**1a**) (C21) is shown in (a), and the only quaternary sp^3^ carbon in strychnine (C7) is shown in (b). Clearly RCSA shifts in traces 5–10 are significantly larger than the change due to ΔΔ*δ*_iso_ seen in traces 0–4, even for C7 (b), which has a very small DFT-computed CSA of 30 ppm. (c) Top traces: C21 of strychnine in weakly (red) and strongly (blue) stretched PMMA gel;^8^ the RCSA value corresponds to the separation between red and blue spectra. Bottom traces: C21 of strychnine with 0% [red, “0” in (a)] and 34.5% PBLG [blue, “10” in (a)]; the actual RCSA value is actually slightly larger than the separation between red and blue spectra after correcting for ΔΔ*δ*_iso_. The red spectra in the PMMA gel and PBLG LC solvent are vertically aligned to illustrate the difference in the RCSA size. Although eleven PBLG concentration were used here to study the trend of chemical shift changes, in practice only three concentrations are needed, *e.g*., samples 0, 4, and 8 are sufficient for RCSA data extraction by the |0–|1–A1 method described below.

**Fig. 3:**
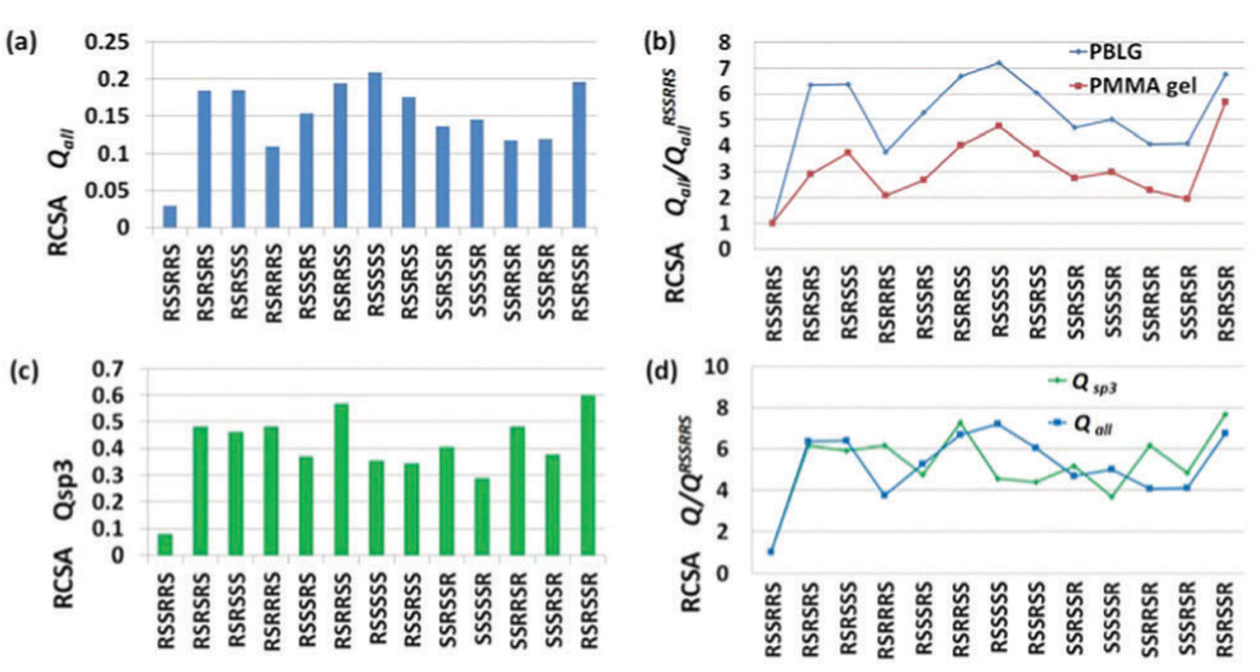
Stereochemical differentiation of strychnine by RCSA data. (a) Differentiation by *Q*-factors using all carbon RCSA data collected in 15.5% PBLG (w/v). (b) Comparison of confidence levels of differentiation between 15.5% PBLG data in (a) (blue) and stretched PMMA gel data in ref. 2*c* (red). (c) Differentiation by *Q*-factors using sp^3^ carbon RCSA data collected in 15.5% PBLG. (d) Comparison of confidence levels of differentiation between using all carbon RCSAs (*Q*_all_) and using only sp^3^ carbon RCSAs (*Q*_sp_^3^) with data collected in 15.5% PBLG.

**Fig. 4:**
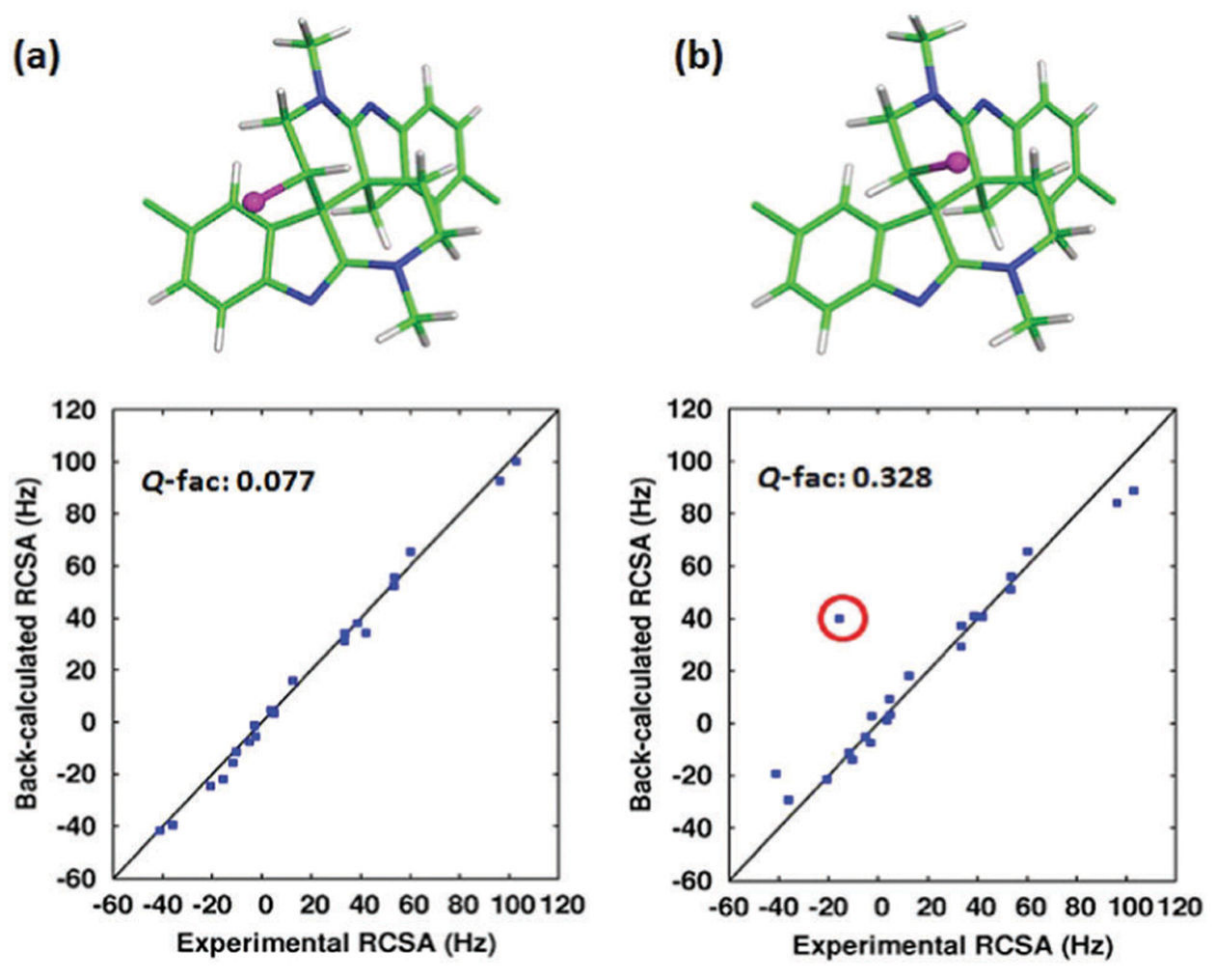
Stereochemical differentiation of caulamidine A using RCSA data collected in PBLG. (a) The structure of caulamidine A reported in ref. 7; (b) the energetically feasible C26-inverted structure. The chlorine atom is represented as a magenta sphere. The outlying point circled in red is the RCSA for the inverted C26.

**Table 1:** RCSA data quality evaluation

	Strychnine (9 sp^2^ and 12 sp^3^ carbons)							Retrorsine^a^ (6 sp^2^ and 10 sp^3^ carbons)		
	Stretched PMMA	11.4% PBLG	12.9% PBLG	15.5% PBLG	18.4% PBLG	22.9% PBLG	34.5% PBLG	Stretched PMMA	12.6% PBLG	15.1% PBLG
GDO	5.56 × 10^−4^	3.47 × 10^−3^	4.67 × 10^−3^	6.58 × 10^−3^	8.58 × 10^−3^	1.21 × 10^−2^	2.05 × 10^−2^	9.76 × 10^−4^	5.04 × 10^−3^	7.63 × 10^−3^
*Q*_all_^b^	0.049	0.032	0.032	0.029	0.028	0.029	0.030	0.104	0.040	0.033
$Q_{{\text{sp}}^{3}}$^c^	0.122	0.060	0.072	0.078	0.074	0.080	0.086	0.184	0.104	0.095
*θ*^d^	12.0°	7.0°	7.4°	7.1°	6.1°	6.6°	7.6°	13.1°	14.0°	4.4°

a Only data from the more rigid part of retrorsine were used for calculations in this table. RCSAs of C23 and C11 are affected by the rotameric rotation of the primary alcohol across the C23–C11 bond, as will be described in a follow-up study, and therefore were omitted here.

b *Q*_all_ represents the *Q*-factor obtained using all available carbon RCSA data.

c *Q*_sp_^3^ represents the *Q*-factor obtained using only sp3 carbon RCSA data.

d The angle *θ* represents the intertensor angle between the alignment tensor determined using all carbon RCSA data and that determined using only sp^3^ carbon RCSA data.
