# Supplementary material for: Enhanced measurement of residual chemical shift anisotropy for small molecule structure elucidation
Source: Chem Commun (Camb). Author manuscript; Available in PMC 2019 Jan 7. (PMC6322199; doi:10.1039/c8cc00552d)
Supplement: supplemental [file NIHMS999720-supplement-supplemental.pdf]

Electronic Supplementary Material (ESI) for Chemical Communications.
This journal is © The Royal Society of Chemistry 2018

Enhanced Measurement of Residual Chemical Shift Anisotropy for Small Molecule Structure Elucidation

Yizhou Liu*[a], Ryan D. Cohen[a], Kirk R. Gustafson[b], Gary E. Martin[a] and R. Thomas Williamson[a]

a. Structure Elucidation Group, Process and Analytical Research and Development, Merck & Co. Inc. 126 East Lincoln Avenue, Rahway, NJ 07065 USA.

b. Molecular Targets Program, Center for Cancer Research, National Cancer Institute, Frederick, Maryland 21702-1201, USA.

Table of Contents

Introduction to the PBLG Liquid Crystal Medium 3
Experimental Procedures 3
Sample Preparation 3
NMR Experiments 3
DFT Calculation 3
Step-by-Step Procedure for NMR Sample Preparation 4
Step-by-Step Procedure for NMR Sample Recovery 5
Summary of Sample Recovery Results 6
Results and Discussion 7
The ^{1}H and ^{13}C spectra under Strong Alignment Conditions 7
Rationale and Limitation of the $\Delta\Delta\delta_{iso}$ Correction Method 8
Correction for $\Delta\Delta\delta_{iso}$ in RCSA measurement 8
Effects of insufficient ^{1}H-decoupling in Strongly Aligned Samples 9
Experimental RCSA Data of Strychnine 10
Experimental RCSA Data of Retrorsine 11
Experimental RCSA Data of Caulamidine A 12
Structure Coordinates and Chemical Shielding Tensors of Strychnine from DFT computation 13
Structure Coordinates of Retrorsine from DFT Geometry Optimization 13
GIAO Chemical Shielding Tensors of Retorsine 15
Structure Coordinates of Caulamidine A from DFT Geometry Optimization 25
GIAO Chemical Shielding Tensors of Caulamidine A 26
Structure Coordinates of the C26-inverted diastereomer of Caulamidine A from DFT Geometry Optimization ... 29
GIAO Chemical Shielding Tensors of the C26-inverted diastereomer of Caulamidine A 30
References 34

Introduction to the PBLG Liquid Crystal Medium

The phase transition and the texture of the cholesteric phase of PBLG have been thoroughly studied by microscopy and X-ray diffraction as described.[1-3] Briefly, at a low [PBLG], the solution is isotropic. When [PBLG] is higher than a critical concentration A, two phases co-exist in equilibrium including a heavier isotropic phase and a polymer-enriched mesophase characterized by a periodic multi-layered supramolecular structure. Increasing [PBLG] beyond A converts more isotropic phase to mesophase with polymer concentrations in both phases staying constant, until [PBLG] reaches a second critical concentration B at which point the whole sample goes into mesophase; at an even higher [PBLG], the supramolecular packing density increases proportionally. The concentration gap between A and B is typically small for high-molecular weight PBLG and in this work we only utilize [PBLG] below A and above B for extraction of RCSA. If a biphasic solution is encountered, which can be revealed by an inhomogeneous ^{2}H spectrum of $CDCl_3$ showing one pair of signals separated by residual quadrupolar coupling (RQC) of several hundred Hz and a singlet or a pair of signals with barely resolved RQC, as described in ref 19 in the main text, a small amount of additional PBLG should be added until only the pair with a large RQC are observed.

Experimental Procedures

Sample Preparation

Strychnine: Strychnine (30mg, MP Biomedicals, Inc.) was dissolved with 600 μL of $CDCl_3$ in a 5 mm NMR tube. Tetramethylsine (TMS) of 4% (v/v) was added for RCSA referencing. Carbon chemical shifts and ^{13}C-^{1}H *J* couplings were measured. Poly-γ-benzyl-L-glutamate (PBLG, 150,000-350,000 Da, Sigma-Aldrich catalog No.: P5136) was successively added to afford weight-to-volume ratios of 2.1%, 4.1%, 6.6%, 8.8%, 11.4%, 12.9%, 15.5%, 18.4%, 22.9%, and 34.5%, and after each addition, carbon chemical shifts were measured, as described in the main text. The ^{2}H spectrum of $CDCl_3$ was taken at each PBLG concentration to measure the residual quadrupolar coupling (RQC). The common method to dissolve PBLG is to briefly and repeatedly centrifuge the NMR tube with interleaved tube inversions, until a clear and homogenous solution was obtained.[4] Solid PBLG is lighter than $CDCl_3$; therefore by repeatedly inverting the NMR tube followed by centrifugation, the undissolved PBLG moves across the sample solution from one end to the other as it dissolves, creating a homogenous PBLG solution. See more details in the section of "Step-by-Step Procedure of NMR Sample Preparation".

Retrorsine: A sample of 10mg retrorsine (Sigma-Aldrich, Inc.) was prepared in the same fashion as for the strychnine sample, but with the successive addition of 0%, 8.1%, 12.6%, and 15.1% PBLG. TMS of 2.5% (v/v) was added for RCSA referencing. Carbon chemical shifts and $CDCl_3$ RQC were measured after each PBLG addition.

Caulamidine A: A sample of 1.6mg caulamidine A was dissolved in 170 μL $CDCl_3$ with 5 μL TMS in a 3mm NMR tube. The material was quantified by external calibration with another sample of 3.6 mg 1,3,5-trimethyoxybenzene (TMB) dissolved in 170 μL $CDCl_3$, through comparison of the ^{1}H integration of caulamidine A or TMB over that of the residual $CHCl_3$ signal. RCSA was measured with I0 of no PBLG, I1 of 6.3% PBLG, and A1 of 13.1% PBLG. Carbon chemical shifts and $CDCl_3$ RQC were measured after each PBLG addition.

NMR Experiments

All NMR data were acquired on a Bruker 600 MHz spectrometer equipped with a helium cooled cryoprobe with the sample temperature maintained at 25 °C. For the measurement of carbon chemical shifts, the standard {^{1}H}-^{13}C experiment was employed with a 90° flip angle, a recycling delay of 2 s, and an acquisition time of 2 s, unless otherwise noted. Strychnine and retrorsine spectra were acquired with 32 and 64 transients, respectively, at all PBLG concentrations. As described in the main text, a higher decoupling field is needed to fully collapse large ^{13}C-^{1}H RDCs as the alignment gets stronger: for strychnine, proton decoupling was conducted using WALTZ16 with a decoupling field of 3125 Hz for the samples of 0%, 2.1%, 4.1%, 6.6%, and 8.8% PBLG in which the samples were isotropic; after alignment was induced at higher PBLG concentrations, proton decoupling was performed using WALTZ16 with a decoupling field of 4167 Hz for the sample of 11.9% PBLG; GARP with a decoupling field of 4167 Hz was used for the samples of 12.9%, 15.5%, and 18.4% PBLG, and GARP with a decoupling field of 8333 Hz was used for the samples of 22.9% and 34.5% PBLG. For retrorsine, proton decoupling was performed using WALTZ16 with a decoupling field of 3125 Hz for samples of 0% and 8.1% PBLG in which the samples were isotropic. GARP with a decoupling field of 4167 Hz was employed for the samples of 12.6% and 15.1% PBLG in which the samples were aligned. Carbon spectra of caulamidine A were collected with 800, 1024, and 3072 transients for 0%, 6.3%, and 13.1% PBLG, respectively. The acquistion time was 0.5 s for caulamidine A with 13.1% PBLG. Proton decoupling was performed with WALTZ16 with a decoupling field of 4167 Hz for 0% and 6.3% PBLG, and GARP with a decoupling field of 4167 Hz for 13.1% PBLG.

DFT Calculation

The density functional theory (DFT)-computed coordinates and chemical shielding tensors of strychnine and its twelve other diastereomers were adopted from a previous publication.[5]

For retrorsine, the initial conformers were collected by combining outputs from three different conformer search programs including ET[6], JG (a Merck in-house version of DG[7]), and OpenEye's OMEGA[8], and were further optimized by DFT in Gaussian 09[9] at the B3LYP/6-31G(d,p) level in the gas phase. The chemical shielding tensors were calculated in Gaussian 09 with the "nmr" keyword at the mPW1PW91/6-31G(d,p) level.

DFT calculation on caulamidine A was performed by a similar procedure as for retrorsine, as reported in Ref 11 of the main text.

Step-by-Step Procedure for NMR Sample Preparation

1. Transfer the sample to a ~5.0 mL glass vial, and dissolve with 0.6 mL $CDCl_3$.
2. Transfer the solution to a 5-mm NMR tube.
3. Weigh the desired amount of PBLG, and roll by hand into a thin rod.

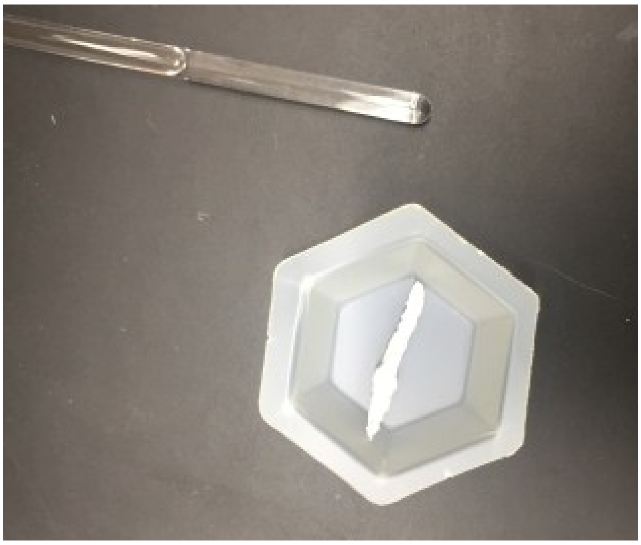

4. Directly insert the rolled PBLG into the NMR tube and cap with a standard NMR tube cap, e.g. New Era P/N NE-310-5 or comparable. To minimize chloroform evaporation, the cap is wrapped with Parafilm M®.

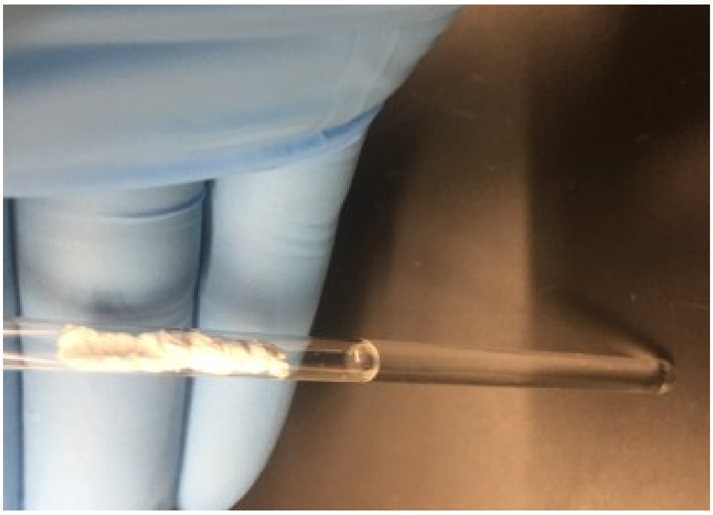

5. Dissolve the PBLG by repeatedly inverting and centrifuging the tube. The PBLG will become a viscous “slug” within the tube. The PBLG “slug” can then be spun back and forth across the sample solution using a low-speed centrifuge, until it fully dissolves. From 20 to 30 inversions may be necessary to fully dissolve the PBLG for a homogenous sample, which takes about 10 mins. A fixed angle rotor is the ideal option for spinning the NMR tube. If a variable angle rotor is used as in our case, it is important to make sure that the NMR tube is shorter than the maximal rotation radius (rMAX) so that the entire sample volume moves back and forth under centrifugation as the NMR tube is inverted.

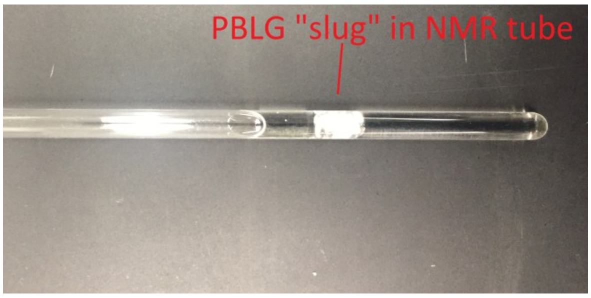

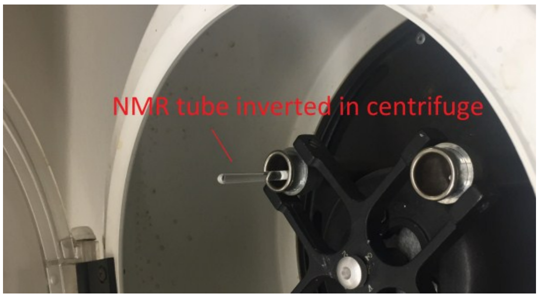

6. When the PBLG is fully dissolved, the solution will be clear.

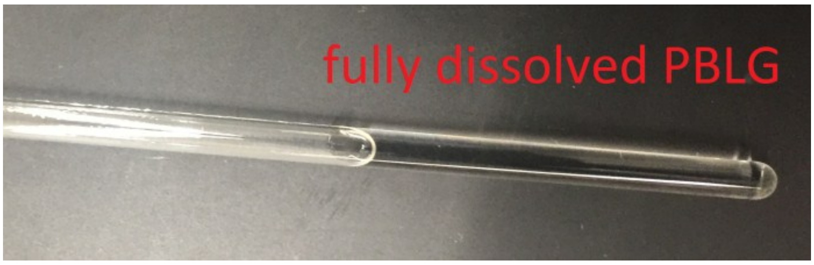

Step-by-Step Procedure for NMR Sample Recovery

1. This sample recovery method is based on insolubility of PBLG in ethyl acetate (EtOAc). Conversely, the sample to be recovered *must* be soluble in EtOAc. If this is not the case, then chromatographic recovery (e.g., size exclusion chromatography) may be feasible.
2. The PBLG solution is quite viscous so it should be spun out of the tube using a centrifuge. First, cut a 0.5 in. piece of 3/16 in. I.D. Nalgene® tubing, which will fit snuggly around the 5 mm NMR tube. Drill a 0.5 cm hole in the top of a 15 mL conical bottom polypropylene centrifuge tube (Fisherbrand, P/N 05-539-12). Pre-rinse the centrifuge tube twice with EtOAc to remove any contaminants (e.g., plasticizer).

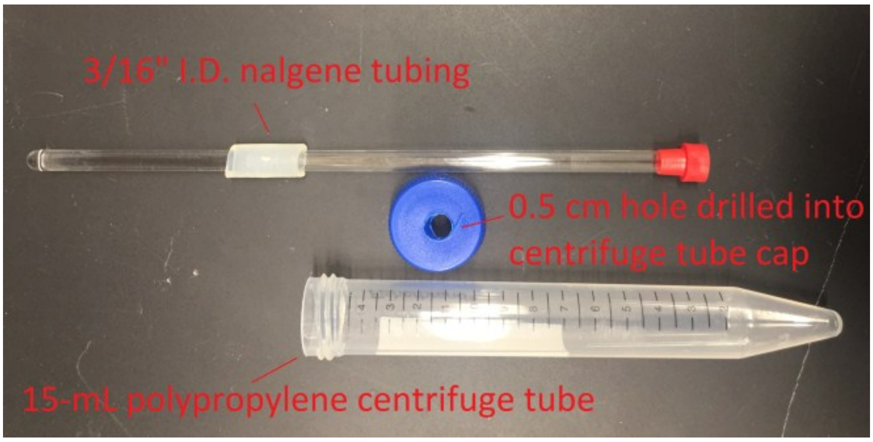

3. Insert the NMR tube into the centrifuge tube upside down and with the Nalgene® tubing “stopper” at the top such that the open end of the NMR tube just touches the beginning of the conical taper. Be sure to leave enough room below the open end of the NMR tube so that the sample solution spun out does not contact the tube opening.

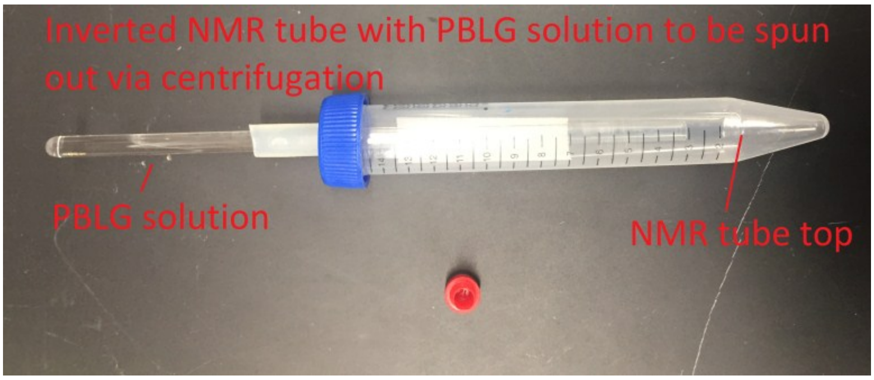

4. Gently spin out the PBLG solution using a centrifuge. Rinse with 1-2 mL $CDCl_3$ followed by EtOAc to recover sample from the walls of the NMR tube, and continue to spin out each time using a centrifuge.
5. Concentrate the solution in the centrifuge tube under a stream of dry N_2 gas. Periodic agitation may be needed as the PBLG may form a film on top of the solution.
6. Add approximately 10 mL of EtOAc to precipitate the PBLG. The precipitation may be aided by vortexing. Further concentrate the solution down to 5 mL, which should fully remove residual $CDCl_3$.

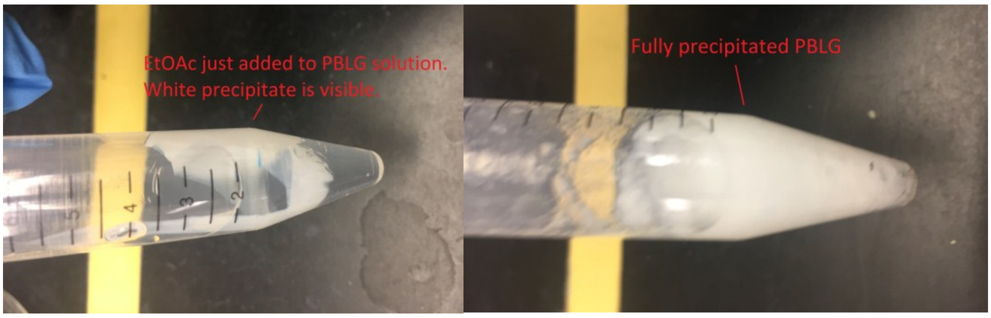

7. Rinse a 1.0 μm PTFE filter (Whatman Puradisc® 25 TF) and 5 mL plastic Luer-lock syringe with EtOAc to remove contaminants.
8. Centrifuge the precipitated solution in the centrifuge tube, and filter the supernatant into a 5 mL glass vial. Repeat by washing the filtrate 1-2 times more with EtOAc to ensure thorough sample recovery.
9. The EtOAc can then be simply removed under a stream of dry N_2 gas.

Summary of Sample Recovery Results

The sample recovery procedure was initially optimized on ibuprofen and then tested for seven additional small molecule compounds: fluconazole, acetanilide, lidocaine, trimethoprim, avobenzone, benzocaine, carbamazepine (all compounds purchased from Sigma-Aldrich as certified reference standards). Recovery was based on the insolubility of PBLG in ethyl acetate. Most small molecules that are soluble in chloroform are also soluble in ethyl acetate. However, in cases where the compound to be recovered is insolube in ethyl acetate, then chromatographic separation is recommended. The key to high recovery is ensuring that the viscous PBLG solution can be fully transferred between the NMR tube and centrifuge tube for precipitation with ethyl acetate. Several rinses with both chloroform and ethyl acetate were found to improve recovery from ~60% to >90%. Fig S1 shows the spectrum of ibuprofen before and after recovery. A small amount of extractable plasticizer (dioctyl phthalate) was also initially observed. This was traced to the plastic syringe barrel and could be minimized by pre-rinsing the syringe with ethyl acetate or by using glass Luer-lock syringes. Simultaneous recovery of all eight tested compounds was found to be greater than 90% as measured by quantitative NMR. For the qNMR measurement, 1,3,5-trimethoxybenzene (TMB) was used as internal standard, and a relaxation delay of 60 s was used with a tip angle of 30°. The 6.1 ppm aromatic signal of TMB did not interfere with any sample component signals and was thus utilized for quantitation. The resolved signals of each compound used for quantitation were as follows: fluconazole, 8.06 ppm, s, 2H; acetanilide, 2.17 ppm, s, 3H; lidocaine, q, 4H; trimethoprim, 6.38 ppm, s, 2H; avobenzone, 7.97 ppm, d, 2H; ibuprofen, 7.21 ppm, d, 2H; benzocaine, d, 6.63 ppm, 2H; carbamazepine, 6.93 ppm, s, 2H.

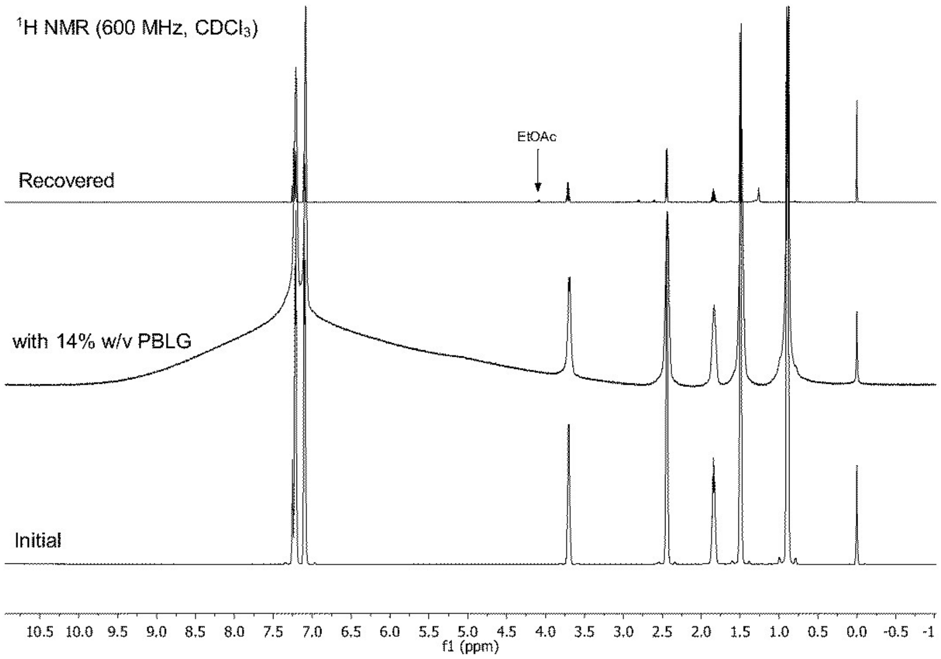

Figure S1. NMR spectra from the initial sample recovery test with ibuprofen. Note that the broad PBLG signal centered around 7.2 ppm completely disappeared after recovery. Although the recovery rate of this initial test was estimated to be only ~60%, the improved procedure achieved a 94.9% recovery rate (Table S1) by fully transferring the viscous PBLG sample from the NMR tube to the centrifugation tube.

Table S1. Sample recovery from PBLG solutions

Compound	MW (g/mol)	Purity (%)	Initial Wt (mg)	Recovered Wt (mg)	Recovery (%)
fluconazole	306.3	99.6	2.54	2.38	93.6
acetanilide	135.2	99.9	2.62	2.40	91.7
lidocaine	234.3	99.8	3.79	3.62	95.6
trimethoprim	290.3	99.8	4.65	4.44	95.5
avobenzone	310.4	99.9	4.52	4.28	94.6
ibuprofen	206.3	99.9	4.43	4.21	94.9
benzocaine	165.2	99.9	4.23	4.02	95.0
carbamazepine	236.3	99.9	3.83	3.81	99.4

Results and Discussion

The ^{1}H and ^{13}C spectra under Strong Alignment Conditions

With strong alignment in the PBLG mesophase, the homonuclear RDCs introduce an extensively coupled network and cause severe line-broadening of the ^{1}H spectrum. As shown in Fig S2a, in going from 0% to 8.1% PBLG, both of which were isotropic based on the absence of $CDCl_3$ RQC (Fig S2b), the increase in ^{1}H line-width was very modest. However, increasing the PBLG concentration to 12.6% caused a transition to the mesophase, as indicated by a $CDCl_3$ RQC of 272.6 Hz (Fig S2b), which was accompanied by substantial ^{1}H line-broadening due to the emergence of RDCs; such broadening was more pronounced with even stronger alignment at 15.1% PBLG ($CDCl_3$ RQC = 406.5 Hz). In contrast, as shown in Fig S2c, in the ^{13}C spectrum both the inhomogeneous broadening and homogenous broadening due to proton spin diffusion can be largely suppressed by proton decoupling, and therefore the line-width increase in PBLG mesophase is much less severe for ^{13}C than for ^{1}H. The sharp ^{13}C resonances faciliate accurate RCSA measurement even at high degrees of alignment. Also note that except for the two broad signals between 132 and 134ppm (Fig S2c expansion to the left), other background ^{13}C signals from the PBLG LC medium is extremely weak, yielding very clean spectra.

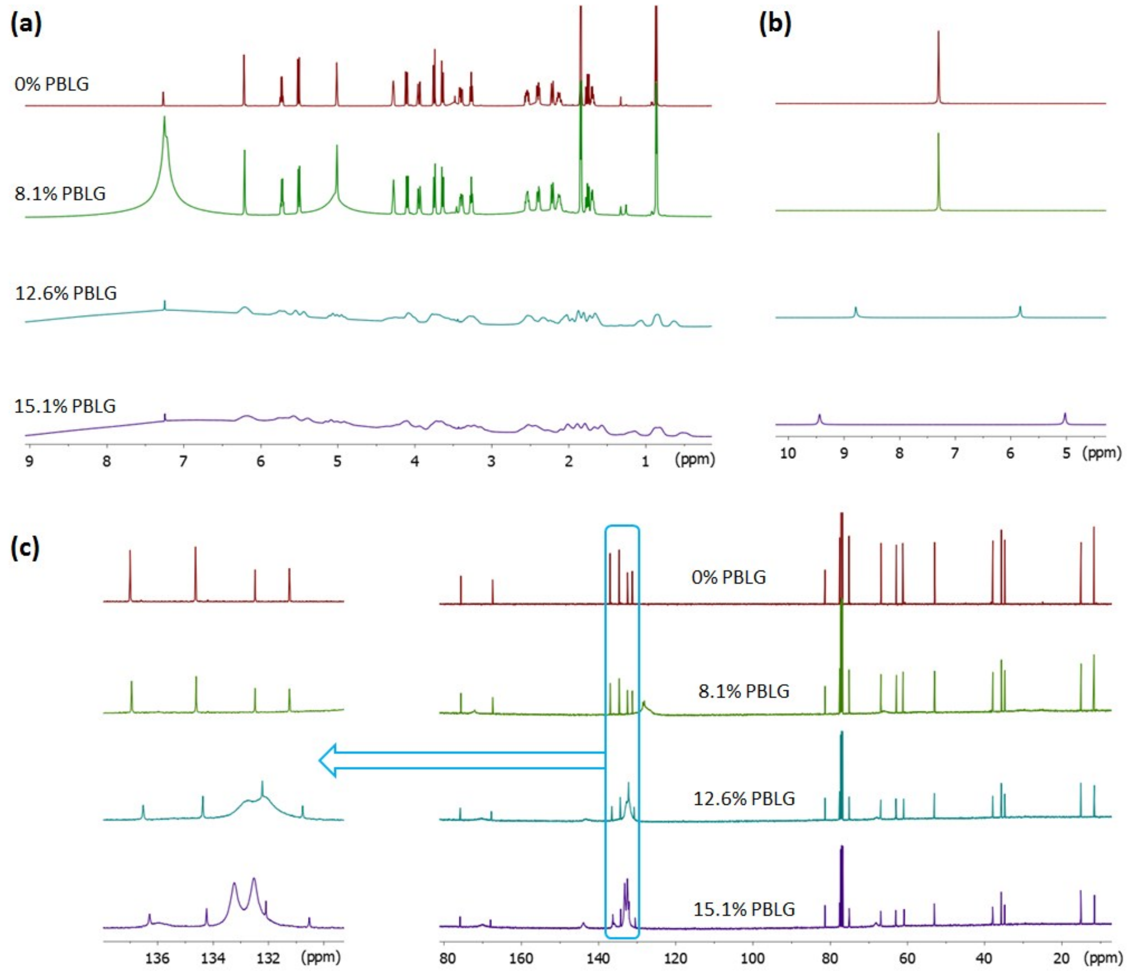

Figure S2. ^{1}H(a) and $^{13}C\{^{1}H\}$(c) spectra of retrorsine and ^{2}H (b) spectrum of $CDCl_3$ at different PBLG concentrations.

Rationale and Limitation of the $\Delta\Delta\delta_{iso}$ Correction Method

Our correction method is based on the premise that the $\Delta\Delta\delta_{iso}$ dependence on PBLG concentration is identical in both phases, such that the $\Delta\Delta\delta_{iso}$ in the mesophase can be corrected for by extrapolating its value from its trend in the isotropic phase. This assumption is reasonable because of two points. First, the analyte can diffuse freely through the highly fluidic mesophase,[1-3] and second, $\Delta\Delta\delta_{iso}$ is determined to the first order by the total surface area of PBLG polymers from averaging all microstates of the analyte with orientational and positional degrees of freedom at the intermolecular interface. An effect not considered, however, is the interference between different surfaces on neighbouring PBLG molecules, which can become relevant with increasing PBLG concentration. This effect can, for instance, reduce the accessible area when two PBLG surfaces are very close or alter the microstates of an analyte trapped between two surfaces. It is noteworthy that the orientational redistribution of PBLG during the phase transition, while generating RCSA, is not expected to cause $\Delta\Delta\delta_{iso}$ by itself, although associated changes in surface interference may. However, as our studies in this work demonstrate, ignoring the interference does not cause significant errors to RCSA measurement.

Correction for $\Delta\Delta\delta_{iso}$ in RCSA measurement

As shown in Fig S3, the TMS-referenced isotropic carbon chemical shift (^{13}C $\Delta\Delta\delta_{iso}$) approximately follows a linear relationship as a function of PBLG concentration when the concentration is below the critical LC forming concentration. As described in the main text, this linear dependence forms the basis for the ^{13}C $\Delta\Delta\delta_{iso}$ correction used in the I0-I1-A1 method for RCSA measurement. Note that the ^{13}C $\Delta\Delta\delta_{iso}$, as shown in Fig S3, is quite small in comparison to ^{13}C RCSA (See Table S1).

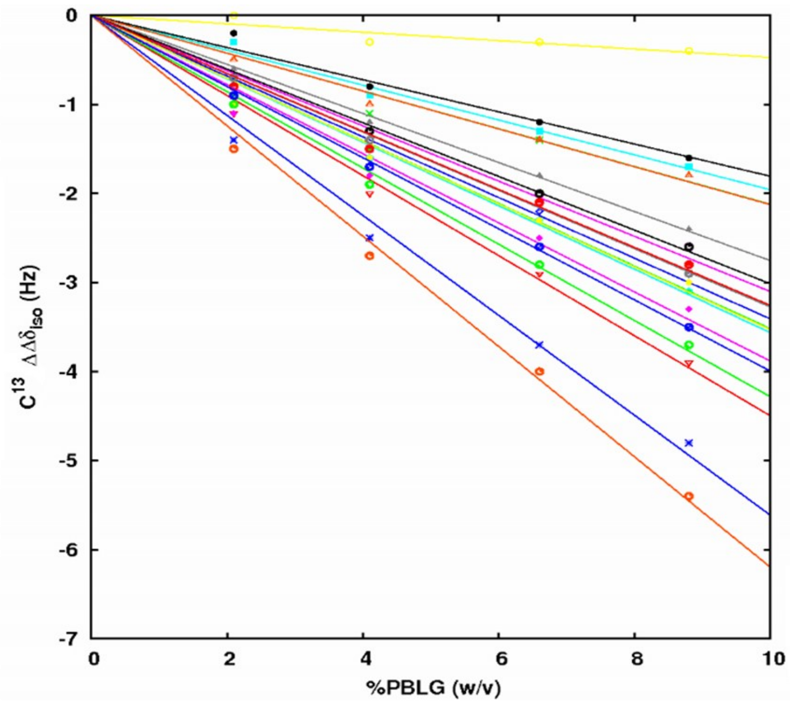

Figure S3. ^{13}C $\Delta\Delta\delta_{iso}$ of strychnine as functions of PBLG concentration. The curves were fitted to y=a*x through linear regression. Curves for all 21 carbons of strychnine are displayed. Data were acquired on a 600 MHz spectrometer. Chemical shifts were internally referenced to TMS.

Effects of insufficient ^1H-decoupling in Strongly Aligned Samples

The potentially large and extensive ^{13}C-^1H RDCs observed for strongly aligned molecules may exceed the default ^1H decoupling capability commonly used for solution NMR experiments. As shown in Fig S4a (top trace), the default ^1H decoupling by WALTZ16 with a decoupling field of 3125 Hz led to the residual multiplet structure for C23 of strychnine aligned in 11.9% PBLG (Refer to Fig 1a in the main text for atomic numbering), whereas increasing the decoupling field to 4167 Hz collapsed the multiplet (Fig S4a, bottom trace); with an even stronger alignment at 12.9% PBLG, C23 showed up again as a residual multiplet with 4167 Hz WALTZ16 (Fig S4b, top trace). Switching from WALTZ16 to GARP yielded better off-resonance decoupling efficiency at the same decoupling power and restored the singlet (Fig S4b, bottom trace).

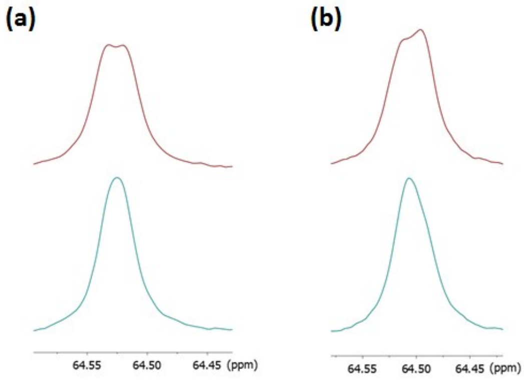

Figure S4. Spectral effects of insufficient ^1H-decoupling in the presence of large RDCs in strongly aligned PBLG mesophase. (a) & (b): Red and blue spectra were acquired with insufficient and sufficient ^1H-decoupling, respectively.

Experimental RCSA Data of Strychnine

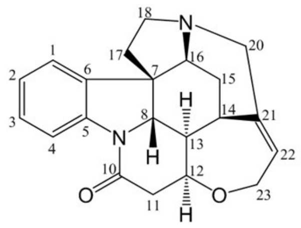

Table S2. Experimental RCSA data (in Hz[a]) of strychnine after correction for $\Delta\Delta\delta_{iso}$ in various PBLG mesophases.

	[PBLG] (w/v)					
	**11.4%**	**12.9%**	**15.5%**	**18.4%**	**22.9%**	**34.5%**
**C10**	26.8	35.8	50	65.4	92.6	158.9
**C5**	29.3	39.5	55.9	73.3	104.9	181.8
**C21**	34.9	46.7	65.1	84.2	116.6	187
**C6**	45.4	61.3	86.6	113.7	N/A[b]	281.2
**C3**	52.2	70.3	99.2	130.3	186.2	324.1
**C22**	23.8	31.6	43.5	55.9	76.3	119
**C2**	45.4	61.1	86	112.7	160.2	275.5
**C1**	67.2	90.5	127.3	166.4	235	396.2
**C4**	59.2	79.7	112.1	146.5	207.8	353.1
**C12**	1.8	2.1	2.5	2.9	3.7	4.8
**C23**	-6.2	-8.6	-14.3	-18	-26.5	-46.8
**C16**	-2.4	-3.5	-5.3	-7.3	-10.5	-18.7
**C8**	-14.3	-19.8	-28.1	-36.9	-52.4	-88.9
**C20**	1.3	2	2.6	0.9	1.9	1.7
**C7**	-3.4	-5.1	-7.5	-10	-14.6	-25.7
**C18**	13.6	18.2	25.2	32.7	45.8	76.5
**C13**	0.4	0.1	-0.6	-1.1	-2.3	-5.4
**C17**	6.4	8.6	11.8	15.4	22	38.3
**C11**	-4.7	-6.8	-9.9	-13.5	-19.6	-36.5
**C14**	2.8	3.6	4.8	6	8.3	14
**C15**	-6.7	-9.4	-13.6	-17.9	-25.5	-43.4
**$CDCl_3$ RQC**	215.5	286.4	394.9	505.6	687.4	1084.2

[a] Values in Hz are based on a spectrometer frequency of 600 MHz. [b] Data not available due to overlap with PBLG benzyl signals.

Experimental RCSA Data of Retrorsine

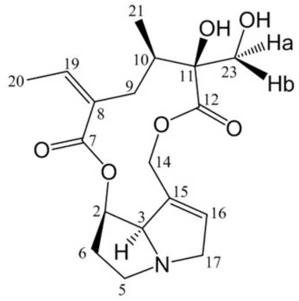

Table S3. Experimental RCSA data (in Hz[a]) of retrorsine after correction for $\Delta\Delta\delta_{iso}$ in PBLG mesophases.

	[PBLG] (w/v)	
	**12.6%**	**15.1%**
**C12**	19.8	29.6
**C7**	54.4	82.4
**C16**	-58.3	-90.4
**C19**	-35.2	-55
**C8**	-39.9	-59.5
**C15**	-71.7	-107.2
**C11**	4.1	7.2
**C3**	9.3	13.1
**C2**	-1.4	-3
**C23**	5.2	9
**C17**	19.5	29.7
**C14**	-21.6	-35.7
**C5**	8.5	12.9
**C9**	4.6	7.5
**C10**	9.9	14.9
**C6**	7.9	11.7
**C20**	6.8	9.9
**C21**	-8.2	-13.1
**$CDCl_3$ RQC**	272.6	406.5

[a] Values in Hz are based on a spectrometer frequency of 600 MHz.

Experimental RCSA Data of Caulamidine A

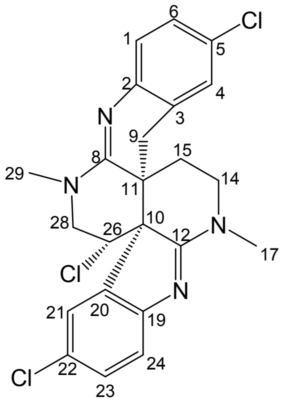

Table S4. Experimental RCSA data (in Hz[a]) of caulamidine A after correction for $\Delta\Delta\delta_{iso}$ in PBLG mesophases.

	[PBLG] (w/v)
	**13.1%**
**C12**	33.5
**C8**	42.1
**C19**	3.6
**C2**	53.3
**C20**	N/A[b]
**C23**	-11.7
**C6**	102.9
**C22**	-3
**C5**	12.5
**C4**	53.6
**C1**	60.1
**C3**	96.2
**C21**	38.7
**C24**	33.7
**C10**	4.6
**C26**	-15.6
**C28**	-41.2
**C14**	-36.2
**C11**	-2.5
**C17**	-20.7
**C29**	-10.3
**C9**	-5
**C15**	4.9
**$CDCl_3$ RQC**	385.7

[a] Values in Hz are based on a spectrometer frequency of 600 MHz.
[b] Resonance of C20 overlaped with the benzyl signals of PBLG and could not be reliably interpreted.

Structure Coordinates and Chemical Shielding Tensors of Strychnine from DFT computation

Refer to supporting information from Ref 5.

Structure Coordinates of Retrorsine from DFT Geometry Optimization

Conformer 1

C	2.234	-0.125	1.162
C	3.684	0.344	0.978
C	4.120	-0.378	-0.305
N	3.438	-1.675	-0.239
C	2.344	-1.613	0.762
C	2.928	-2.238	-1.494
C	1.530	-2.672	-1.155
C	1.188	-2.321	0.088
O	1.345	0.537	0.228
C	1.050	1.841	0.478
C	0.062	2.370	-0.516
O	1.534	2.463	1.405
C	0.142	3.636	-0.965
C	1.145	4.696	-0.626
C	-1.030	1.418	-0.955
C	-2.024	1.102	0.200
C	-0.089	-2.691	0.775
O	-1.043	-1.586	0.853
C	-1.797	-1.384	-0.232
C	-2.790	-0.222	-0.066
O	-1.727	-2.032	-1.261
C	-2.973	2.288	0.426
O	-3.544	-0.145	-1.273
C	-3.785	-0.595	1.052
O	-4.413	-1.829	0.754
H	2.628	-2.153	1.683
H	1.920	4.342	0.052
H	1.606	5.086	-1.542
H	0.643	5.546	-0.147
H	-3.684	2.370	-0.401
H	-3.538	2.197	1.358
H	-2.401	3.219	0.483
H	4.271	-0.008	1.833
H	3.772	1.429	0.932
H	5.209	-0.494	-0.364
H	3.803	0.197	-1.192
H	3.540	-3.082	-1.848
H	2.928	-1.494	-2.310
H	-0.569	0.497	-1.322
H	-1.586	1.851	-1.792
H	0.082	-2.955	1.822
H	-0.576	-3.523	0.264
H	-4.521	0.213	1.154
H	-3.263	-0.716	2.005
H	1.853	0.035	2.173
H	-1.442	0.937	1.115
H	0.890	-3.211	-1.846
H	-0.614	3.934	-1.692
H	-3.113	-0.766	-1.888
H	-4.758	-1.720	-0.146

Conformer 2

C	2.277	-0.127	1.121
C	3.718	0.335	0.867
C	4.096	-0.408	-0.422
N	3.428	-1.707	-0.297
C	2.364	-1.621	0.735
C	2.882	-2.307	-1.519
C	1.486	-2.710	-1.138
C	1.180	-2.323	0.104
O	1.351	0.529	0.222
C	1.056	1.830	0.491
C	0.045	2.367	-0.474
O	1.567	2.444	1.410
C	0.092	3.650	-0.878

C	1.070	4.725	-0.511
C	-1.030	1.410	-0.942
C	-2.063	1.092	0.178
C	-0.090	-2.647	0.826
O	-1.037	-1.530	0.845
C	-1.812	-1.397	-0.237
C	-2.804	-0.236	-0.117
O	-1.798	-2.139	-1.203
C	-3.015	2.278	0.386
O	-3.499	-0.138	-1.357
C	-3.849	-0.640	0.970
O	-5.060	0.060	0.802
H	2.667	-2.155	1.653
H	1.884	4.361	0.113
H	1.476	5.190	-1.417
H	0.556	5.522	0.042
H	-3.616	2.450	-0.513
H	-3.703	2.111	1.216
H	-2.437	3.184	0.588
H	4.343	-0.004	1.701
H	3.805	1.419	0.800
H	5.181	-0.519	-0.533
H	3.731	0.149	-1.301
H	3.475	-3.172	-1.856
H	2.876	-1.592	-2.361
H	-0.553	0.490	-1.292
H	-1.560	1.837	-1.798
H	0.090	-2.846	1.885
H	-0.587	-3.504	0.370
H	-3.462	-0.423	1.970
H	-4.016	-1.729	0.906
H	1.944	0.044	2.147
H	-1.514	0.920	1.112
H	0.822	-3.255	-1.800
H	-0.678	3.954	-1.588
H	-3.244	-0.928	-1.866
H	-5.203	0.080	-0.159

Conformer 3

C	2.233	-0.123	1.158
C	3.683	0.343	0.969
C	4.111	-0.376	-0.317
N	3.434	-1.676	-0.244
C	2.343	-1.612	0.760
C	2.920	-2.243	-1.496
C	1.524	-2.677	-1.153
C	1.186	-2.322	0.091
O	1.341	0.537	0.227
C	1.053	1.845	0.476
C	0.060	2.376	-0.510
O	1.549	2.465	1.397
C	0.130	3.647	-0.946
C	1.127	4.711	-0.601
C	-1.030	1.423	-0.952
C	-2.018	1.098	0.203
C	-0.086	-2.700	0.781
O	-1.048	-1.595	0.861
C	-1.802	-1.387	-0.237
C	-2.785	-0.221	-0.076
O	-1.752	-2.072	-1.241
C	-2.979	2.267	0.452
O	-3.533	-0.122	-1.275
C	-3.788	-0.579	1.058
O	-3.260	-0.475	2.365
H	2.629	-2.152	1.681
H	1.926	4.347	0.044
H	1.556	5.137	-1.516
H	0.627	5.537	-0.078
H	-3.691	2.365	-0.374
H	-3.536	2.134	1.383
H	-2.418	3.203	0.534
H	4.274	-0.011	1.821
H	3.772	1.428	0.925
H	5.200	-0.489	-0.386

H 3.783 0.197 -1.200
H 3.531 -3.089 -1.849
H 2.918 -1.501 -2.315
H -0.566 0.505 -1.326
H -1.589 1.857 -1.787
H 0.088 -2.961 1.827
H -0.571 -3.532 0.270
H -4.198 -1.580 0.851
H -4.618 0.127 0.989
H 1.856 0.037 2.171
H -1.443 0.930 1.119
H 0.883 -3.219 -1.840
H -0.633 3.948 -1.664
H -3.210 -0.829 -1.857
H -2.470 -1.032 2.401

GIAO Chemical Shielding Tensors of Retorsine

Conformer 1

SCF GIAO Magnetic shielding tensor (ppm):
1 C Isotropic = 118.2971 Anisotropy = 35.4300
XX= 128.8877 YX= -4.3810 ZX= 9.1550
XY= -8.1191 YY= 120.7654 ZY= -16.8622
XZ= 13.1827 YZ= -16.7873 ZZ= 105.2382
Eigenvalues: 93.3401 119.6340 141.9171
2 C Isotropic = 160.0854 Anisotropy = 29.8468
XX= 171.9743 YX= 13.6094 ZX= -10.9528
XY= 9.3159 YY= 154.7588 ZY= 5.7070
XZ= -13.8194 YZ= 13.6960 ZZ= 153.5230
Eigenvalues: 136.4114 163.8614 179.9832
3 C Isotropic = 143.1101 Anisotropy = 60.7200
XX= 131.3560 YX= 26.3673 ZX= 4.7023
XY= 26.3498 YY= 169.4999 ZY= 3.9217
XZ= -3.3666 YZ= 8.5922 ZZ= 128.4744
Eigenvalues: 117.4121 128.3281 183.5901
4 N Isotropic = 209.3943 Anisotropy = 68.4081
XX= 210.6918 YX= 54.9610 ZX= -31.7449
XY= 49.1303 YY= 189.3655 ZY= 32.7465
XZ= -36.6052 YZ= 44.4971 ZZ= 228.1257
Eigenvalues: 121.8978 251.2855 254.9997
5 C Isotropic = 116.7926 Anisotropy = 44.5595
XX= 138.2314 YX= 4.9414 ZX= -18.3505
XY= 12.7920 YY= 106.8557 ZY= 15.0610
XZ= -17.7027 YZ= 8.4596 ZZ= 105.2907
Eigenvalues: 86.9275 116.9514 146.4990
6 C Isotropic = 133.1322 Anisotropy = 54.4698
XX= 127.1804 YX= 16.8285 ZX= 20.2505
XY= 19.2152 YY= 125.6598 ZY= 11.8360
XZ= 18.3803 YZ= 16.7535 ZZ= 146.5565
Eigenvalues: 107.9782 121.9730 169.4454
7 C Isotropic = 56.7599 Anisotropy = 122.1683
XX= -3.8471 YX= -61.1908 ZX= -9.5190
XY= -61.7554 YY= 95.1731 ZY= -46.2646
XZ= 9.7357 YZ= -16.0435 ZZ= 78.9536
Eigenvalues: -34.9057 66.9799 138.2054
8 C Isotropic = 65.6832 Anisotropy = 125.4314
XX= 8.2118 YX= -65.1668 ZX= 2.5891
XY= -76.0752 YY= 109.5721 ZY= -11.5871
XZ= -21.2852 YZ= -32.6616 ZZ= 79.2659
Eigenvalues: -31.1427 78.8882 149.3042
9 O Isotropic = 127.7801 Anisotropy = 127.2130
XX= 130.0498 YX= 38.2442 ZX= -90.7903
XY= -34.3119 YY= 212.2565 ZY= -55.0787
XZ= -143.5551 YZ= 58.2208 ZZ= 41.0340
Eigenvalues: -39.8222 210.5738 212.5888
10 C Isotropic = 32.7746 Anisotropy = 81.3721
XX= 49.0285 YX= 44.3055 ZX= -29.3964
XY= 65.6309 YY= -15.2297 ZY= 29.3188
XZ= -14.9342 YZ= 5.3260 ZZ= 64.5251
Eigenvalues: -52.6081 63.9093 87.0227

11 C Isotropic = 63.7546 Anisotropy = 119.2960
XX= 32.1379 YX= -25.4996 ZX= -79.4651
XY= -26.0448 YY= 80.4041 ZY= 4.9857
XZ= -73.0142 YZ= 12.5024 ZZ= 78.7218
Eigenvalues: -26.6044 74.5829 143.2853
12 O Isotropic = -79.1781 Anisotropy = 584.1643
XX= 89.5452 YX= 94.7760 ZX= -228.4047
XY= 102.2926 YY= -214.6684 ZY= -131.7300
XZ= -262.3055 YZ= -186.2744 ZZ= -112.4111
Eigenvalues: -347.4206 -200.3784 310.2648
13 C Isotropic = 57.9259 Anisotropy = 146.7471
XX= 30.5599 YX= -17.9664 ZX= -93.3368
XY= -16.8519 YY= 68.6785 ZY= 26.4992
XZ= -89.1304 YZ= 18.7650 ZZ= 74.5393
Eigenvalues: -41.2977 59.3180 155.7573
14 C Isotropic = 178.9569 Anisotropy = 22.8550
XX= 186.9880 YX= 13.4017 ZX= -3.8940
XY= 9.4725 YY= 176.0073 ZY= 8.2432
XZ= -3.8746 YZ= 5.6287 ZZ= 173.8755
Eigenvalues: 163.0082 179.6690 194.1936
15 C Isotropic = 156.6956 Anisotropy = 20.7363
XX= 165.1452 YX= 8.3580 ZX= -1.5766
XY= 9.5430 YY= 155.3537 ZY= -0.8278
XZ= -1.2726 YZ= 0.9439 ZZ= 149.5879
Eigenvalues: 148.9647 150.6024 170.5198
16 C Isotropic = 156.6988 Anisotropy = 24.6846
XX= 161.4541 YX= 11.9949 ZX= 1.8705
XY= 13.3433 YY= 159.2161 ZY= 3.0310
XZ= 3.0429 YZ= -3.8115 ZZ= 149.4262
Eigenvalues: 146.3483 150.5929 173.1552
17 C Isotropic = 133.2269 Anisotropy = 54.6546
XX= 143.8274 YX= -25.0471 ZX= -10.0829
XY= -22.0258 YY= 147.9441 ZY= -10.2510
XZ= -6.3037 YZ= -12.7796 ZZ= 107.9093
Eigenvalues: 99.4214 130.5961 169.6633
18 O Isotropic = 145.1475 Anisotropy = 165.0444
XX= 192.1643 YX= 69.1155 ZX= -23.9530
XY= 114.6272 YY= 95.0373 ZY= -33.4664
XZ= 79.3026 YZ= -146.8844 ZZ= 148.2409
Eigenvalues: -12.9379 193.2033 255.1771
19 C Isotropic = 21.1367 Anisotropy = 97.1355
XX= 9.6210 YX= 62.5983 ZX= -38.3997
XY= 46.7472 YY= 39.5511 ZY= 10.0446
XZ= -59.1471 YZ= 25.8512 ZZ= 14.2378
Eigenvalues: -64.2006 41.7169 85.8936
20 C Isotropic = 111.9023 Anisotropy = 17.1007
XX= 117.6620 YX= 5.4673 ZX= 11.6103
XY= 7.6885 YY= 111.3355 ZY= -10.6250
XZ= 7.2451 YZ= -15.1541 ZZ= 106.7094
Eigenvalues: 91.0019 121.4022 123.3027
21 O Isotropic = -47.8528 Anisotropy = 591.1316
XX= 59.4301 YX= 222.8414 ZX= -157.8662
XY= 211.9019 YY= 44.2697 ZY= -133.0928
XZ= -187.6291 YZ= -123.3599 ZZ= -247.2581
Eigenvalues: -328.1711 -161.6221 346.2349
22 C Isotropic = 182.4629 Anisotropy = 19.4339
XX= 184.6538 YX= -9.5407 ZX= -4.6308
XY= -12.1680 YY= 183.1809 ZY= 1.0903
XZ= -4.4184 YZ= -1.7143 ZZ= 179.5541
Eigenvalues: 171.5825 180.3874 195.4189
23 O Isotropic = 307.1129 Anisotropy = 62.0687
XX= 305.6724 YX= -39.6522 ZX= -4.5172
XY= -40.3214 YY= 297.1139 ZY= 3.0364
XZ= -9.5672 YZ= 24.0106 ZZ= 318.5525
Eigenvalues: 260.6609 312.1858 348.4921
24 C Isotropic = 129.7327 Anisotropy = 41.1517
XX= 128.7513 YX= 21.9627 ZX= -4.8273
XY= 18.7411 YY= 142.5278 ZY= 1.6607
XZ= -1.4140 YZ= 5.9407 ZZ= 117.9190
Eigenvalues: 110.9148 121.1161 157.1672
25 O Isotropic = 318.5106 Anisotropy = 43.1688
XX= 293.9088 YX= -24.8363 ZX= -3.1320
XY= 6.0107 YY= 314.4310 ZY= -6.0603
XZ= 7.4428 YZ= 5.6579 ZZ= 347.1919

Eigenvalues: 290.1797 318.0623 347.2898
26 H Isotropic = 27.4326 Anisotropy = 7.3912
XX= 25.8409 YX= -0.1994 ZX= 0.1842
XY= -0.5248 YY= 26.7574 ZY= -3.5170
XZ= -0.3177 YZ= -4.1971 ZZ= 29.6997
Eigenvalues: 24.0377 25.9001 32.3601
27 H Isotropic = 28.9206 Anisotropy = 6.1901
XX= 28.7809 YX= 0.2834 ZX= 4.3255
XY= 1.2030 YY= 30.7099 ZY= -1.9655
XZ= 3.5760 YZ= -3.7360 ZZ= 27.2709
Eigenvalues: 23.0512 30.6633 33.0473
28 H Isotropic = 30.3452 Anisotropy = 9.9574
XX= 29.2209 YX= 3.7828 ZX= -3.7468
XY= 2.6346 YY= 30.3746 ZY= -3.3049
XZ= -2.4484 YZ= -3.6743 ZZ= 31.4401
Eigenvalues: 26.5257 27.5265 36.9835
29 H Isotropic = 30.0065 Anisotropy = 9.6755
XX= 27.7899 YX= -1.2312 ZX= -0.8309
XY= -1.6802 YY= 34.9670 ZY= 2.8855
XZ= -1.1556 YZ= 3.5063 ZZ= 27.2626
Eigenvalues: 25.9820 27.5806 36.4568
30 H Isotropic = 30.8014 Anisotropy = 8.7591
XX= 32.6088 YX= -2.5889 ZX= 4.0206
XY= -3.3539 YY= 30.7253 ZY= -1.3191
XZ= 3.7964 YZ= -0.4276 ZZ= 29.0701
Eigenvalues: 26.3231 29.4403 36.6408
31 H Isotropic = 31.0668 Anisotropy = 10.5702
XX= 30.4877 YX= -0.9308 ZX= -5.8803
XY= -2.0244 YY= 30.0950 ZY= 2.0915
XZ= -5.7464 YZ= 1.3932 ZZ= 32.6177
Eigenvalues: 25.6425 29.4443 38.1136
32 H Isotropic = 30.7093 Anisotropy = 8.4979
XX= 31.0901 YX= 1.6673 ZX= -1.3754
XY= 1.9322 YY= 35.6413 ZY= 1.7889
XZ= -2.2208 YZ= 1.6691 ZZ= 25.3965
Eigenvalues: 24.4506 31.3028 36.3745
33 H Isotropic = 29.8527 Anisotropy = 10.7498
XX= 31.9706 YX= -0.1168 ZX= 5.1663
XY= -0.7271 YY= 25.5523 ZY= 0.4959
XZ= 4.8420 YZ= 1.3872 ZZ= 32.0352
Eigenvalues: 25.0620 27.4769 37.0192
34 H Isotropic = 29.0516 Anisotropy = 8.6617
XX= 29.5351 YX= 1.3771 ZX= 0.9215
XY= 1.1464 YY= 34.0729 ZY= 2.3774
XZ= -0.7139 YZ= 2.0898 ZZ= 23.5467
Eigenvalues: 23.0888 29.2399 34.8260
35 H Isotropic = 28.6371 Anisotropy = 11.0472
XX= 35.5824 YX= 0.3968 ZX= -2.0556
XY= 2.3836 YY= 26.0296 ZY= 0.7912
XZ= -1.4169 YZ= 0.8675 ZZ= 24.2993
Eigenvalues: 23.5834 26.3260 36.0019
36 H Isotropic = 29.2178 Anisotropy = 6.7846
XX= 27.4620 YX= 0.5461 ZX= -1.7716
XY= 0.9455 YY= 29.9447 ZY= -3.8165
XZ= -2.5735 YZ= -2.0448 ZZ= 30.2467
Eigenvalues: 26.0310 27.8816 33.7409
37 H Isotropic = 27.7868 Anisotropy = 10.7887
XX= 26.7203 YX= -2.1366 ZX= -1.4189
XY= -3.6322 YY= 28.9633 ZY= 5.3876
XZ= -1.6083 YZ= 5.4187 ZZ= 27.6768
Eigenvalues: 22.6830 25.6982 34.9792
38 H Isotropic = 28.3701 Anisotropy = 7.0221
XX= 26.3535 YX= -0.7453 ZX= -0.0111
XY= 1.3738 YY= 25.7733 ZY= 0.0682
XZ= -1.3352 YZ= -0.1116 ZZ= 32.9834
Eigenvalues: 25.6267 26.4321 33.0515
39 H Isotropic = 29.3345 Anisotropy = 5.2458
XX= 30.7822 YX= -0.9214 ZX= -0.8953
XY= -1.5080 YY= 31.7184 ZY= 0.6418
XZ= -0.8913 YZ= 1.6040 ZZ= 25.5029
Eigenvalues: 25.2213 29.9505 32.8317
40 H Isotropic = 30.1175 Anisotropy = 5.4567
XX= 29.0819 YX= 1.2528 ZX= 1.2471
XY= 1.5645 YY= 29.2917 ZY= -2.4531

XZ=   1.3391   YZ=   -3.1137   ZZ=   31.9790
Eigenvalues: 26.2767   30.3206   33.7553
41 H   Isotropic =   27.6312   Anisotropy =   7.1965
XX=   27.0602   YX=   -1.0955   ZX=   -0.0138
XY=   -1.3818   YY=   27.1075   ZY=   -5.4016
XZ=   -0.2040   YZ=   -3.2823   ZZ=   28.7260
Eigenvalues: 23.2236   27.2412   32.4288
42 H   Isotropic =   26.1401   Anisotropy =   6.0424
XX=   26.2640   YX=   -0.2042   ZX=   -0.5374
XY=   0.4146   YY=   30.1643   ZY=   1.2232
XZ=   -0.4419   YZ=   -1.0036   ZZ=   21.9919
Eigenvalues: 21.9348   26.3172   30.1683
43 H   Isotropic =   27.9522   Anisotropy =   6.4638
XX=   28.3437   YX=   1.0286   ZX=   -3.2072
XY=   -0.0796   YY=   31.8723   ZY=   1.7325
XZ=   -3.6468   YZ=   1.8288   ZZ=   23.6405
Eigenvalues: 21.5234   30.0717   32.2614
44 H   Isotropic =   28.0707   Anisotropy =   2.7206
XX=   27.7599   YX=   1.1321   ZX=   0.3312
XY=   2.0490   YY=   26.5927   ZY=   -0.0970
XZ=   -0.0438   YZ=   -0.4754   ZZ=   29.8596
Eigenvalues: 25.4592   28.8685   29.8845
45 H   Isotropic =   26.9790   Anisotropy =   3.9103
XX=   26.6164   YX=   -0.6763   ZX=   0.0121
XY=   -0.8427   YY=   24.9036   ZY=   -1.4197
XZ=   -0.5692   YZ=   -0.3239   ZZ=   29.4169
Eigenvalues: 24.4472   26.9038   29.5859
46 H   Isotropic =   30.0850   Anisotropy =   5.7304
XX=   32.1494   YX=   -0.8587   ZX=   2.9021
XY=   -0.6938   YY=   28.9185   ZY=   -0.0492
XZ=   2.1272   YZ=   -2.2822   ZZ=   29.1871
Eigenvalues: 27.4310   28.9187   33.9053
47 H   Isotropic =   25.0793   Anisotropy =   4.5864
XX=   25.1002   YX=   -2.0285   ZX=   -2.8323
XY=   -1.0981   YY=   23.7421   ZY=   0.1784
XZ=   0.6535   YZ=   3.1037   ZZ=   26.3956
Eigenvalues: 22.5148   24.5862   28.1369
48 H   Isotropic =   25.6546   Anisotropy =   8.0779
XX=   24.2297   YX=   3.0571   ZX=   -0.7005
XY=   2.8270   YY=   29.3166   ZY=   -1.3486
XZ=   -0.4442   YZ=   -1.8585   ZZ=   23.4174
Eigenvalues: 22.7991   23.1248   31.0399
49 H   Isotropic =   28.2712   Anisotropy =   19.3048
XX=   25.4550   YX=   -6.4159   ZX=   -2.7531
XY=   -6.5927   YY=   28.0821   ZY=   8.3592
XZ=   -2.6760   YZ=   9.1227   ZZ=   31.2766
Eigenvalues: 18.6188   25.0538   41.1411
50 H   Isotropic =   30.3778   Anisotropy =   14.9387
XX=   29.2028   YX=   5.8264   ZX=   6.1579
XY=   4.4445   YY=   24.7521   ZY=   -1.2733
XZ=   5.6466   YZ=   -2.9193   ZZ=   37.1785
Eigenvalues: 19.8436   30.9529   40.3369

Conformer 2

1 C   Isotropic =   118.8594   Anisotropy =   35.2074
XX=   130.6916   YX=   -4.7833   ZX=   8.2567
XY=   -8.5825   YY=   121.2657   ZY=   -16.5420
XZ=   11.9145   YZ=   -16.5582   ZZ=   104.6208
Eigenvalues: 93.6564   120.5908   142.3310
2 C   Isotropic =   160.0414   Anisotropy =   29.4846
XX=   170.7418   YX=   13.6603   ZX=   -11.6768
XY=   8.9537   YY=   155.3641   ZY=   4.8087
XZ=   -14.8102   YZ=   12.9699   ZZ=   154.0183
Eigenvalues: 136.8415   163.5849   179.6978
3 C   Isotropic =   142.9119   Anisotropy =   60.7144
XX=   130.6802   YX=   26.7596   ZX=   4.2607
XY=   26.5195   YY=   169.5664   ZY=   2.1789
XZ=   -4.7645   YZ=   6.9379   ZZ=   128.4891
Eigenvalues: 116.6897   128.6578   183.3882
4 N   Isotropic =   209.6960   Anisotropy =   67.6410
XX=   208.1492   YX=   54.7160   ZX=   -32.3123
XY=   48.5990   YY=   191.9383   ZY=   32.6903
XZ=   -37.0497   YZ=   43.9475   ZZ=   229.0005
Eigenvalues: 122.6532   251.6448   254.7900

5 C Isotropic = 116.6724 Anisotropy = 45.3038
XX= 137.5352 YX= 5.0184 ZX= -19.6552
XY= 12.3234 YY= 106.4946 ZY= 15.0144
XZ= -18.9483 YZ= 9.4646 ZZ= 105.9875
Eigenvalues: 86.0821 117.0601 146.8749
6 C Isotropic = 133.2056 Anisotropy = 54.4252
XX= 128.7701 YX= 17.7976 ZX= 20.0731
XY= 19.4105 YY= 126.1290 ZY= 12.3530
XZ= 18.5875 YZ= 16.6153 ZZ= 144.7177
Eigenvalues: 108.4809 121.6469 169.4890
7 C Isotropic = 56.6010 Anisotropy = 122.6951
XX= -6.1856 YX= -60.5974 ZX= -7.0676
XY= -59.7375 YY= 96.3436 ZY= -45.4914
XZ= 12.5189 YZ= -15.4494 ZZ= 79.6451
Eigenvalues: -34.9362 66.3415 138.3978
8 C Isotropic = 65.4763 Anisotropy = 125.6448
XX= 4.8773 YX= -63.5870 ZX= 5.5089
XY= -75.3029 YY= 110.8925 ZY= -10.6153
XZ= -17.9031 YZ= -32.0905 ZZ= 80.6591
Eigenvalues: -31.5183 78.7077 149.2395
9 O Isotropic = 127.8892 Anisotropy = 128.1077
XX= 123.3972 YX= 40.3025 ZX= -91.2884
XY= -34.3894 YY= 212.9762 ZY= -51.1552
XZ= -145.9159 YZ= 56.0550 ZZ= 47.2941
Eigenvalues: -39.2660 209.6392 213.2943
10 C Isotropic = 32.8605 Anisotropy = 80.9091
XX= 47.8476 YX= 44.4676 ZX= -28.0970
XY= 66.4994 YY= -15.7500 ZY= 26.3399
XZ= -13.3548 YZ= 3.1438 ZZ= 66.4837
Eigenvalues: -52.4382 64.2197 86.7999
11 C Isotropic = 64.2462 Anisotropy = 119.8926
XX= 33.0840 YX= -23.5184 ZX= -80.4378
XY= -24.1947 YY= 78.7925 ZY= 3.6902
XZ= -73.9459 YZ= 11.4479 ZZ= 80.8621
Eigenvalues: -25.9473 74.5113 144.1746
12 O Isotropic = -77.5665 Anisotropy = 582.9867
XX= 75.1112 YX= 91.5048 ZX= -233.0669
XY= 101.5533 YY= -208.1002 ZY= -136.9978
XZ= -266.8039 YZ= -187.9726 ZZ= -99.7105
Eigenvalues: -344.8050 -198.9858 311.0913
13 C Isotropic = 56.7479 Anisotropy = 148.4275
XX= 28.8216 YX= -16.9946 ZX= -94.3489
XY= -14.8076 YY= 64.7743 ZY= 24.5862
XZ= -89.9864 YZ= 17.9156 ZZ= 76.6478
Eigenvalues: -42.4863 57.0305 155.6996
14 C Isotropic = 178.7488 Anisotropy = 23.0864
XX= 186.2368 YX= 14.0276 ZX= -3.7861
XY= 10.0923 YY= 175.7273 ZY= 7.8619
XZ= -4.1798 YZ= 5.0863 ZZ= 174.2823
Eigenvalues: 162.7990 179.3077 194.1397
15 C Isotropic = 155.6322 Anisotropy = 21.2630
XX= 164.5700 YX= 8.8664 ZX= -2.2023
XY= 9.3398 YY= 153.5687 ZY= 0.3526
XZ= -1.6019 YZ= 0.4293 ZZ= 148.7579
Eigenvalues: 147.2676 149.8213 169.8075
16 C Isotropic = 153.5745 Anisotropy = 28.5469
XX= 157.1999 YX= 15.0925 ZX= 2.7049
XY= 18.1820 YY= 154.4839 ZY= 1.9844
XZ= 1.9976 YZ= -3.2723 ZZ= 149.0396
Eigenvalues: 138.7328 149.3849 172.6058
17 C Isotropic = 133.6778 Anisotropy = 55.0343
XX= 143.6852 YX= -25.9272 ZX= -9.9338
XY= -22.6847 YY= 147.3987 ZY= -13.3581
XZ= -5.8852 YZ= -14.9142 ZZ= 109.9495
Eigenvalues: 98.9226 131.7435 170.3674
18 O Isotropic = 140.7118 Anisotropy = 165.7172
XX= 190.7615 YX= 59.7338 ZX= -34.1211
XY= 112.4143 YY= 74.3456 ZY= -35.3791
XZ= 60.6729 YZ= -143.1421 ZZ= 157.0284
Eigenvalues: -14.4583 185.4039 251.1899
19 C Isotropic = 21.3655 Anisotropy = 97.2251
XX= 8.9162 YX= 60.0065 ZX= -43.3441
XY= 43.2360 YY= 42.3201 ZY= 10.1046
XZ= -61.9964 YZ= 22.8157 ZZ= 12.8603

Eigenvalues: -64.8215 42.7359 86.1823
20 C Isotropic = 115.0263 Anisotropy = 24.8418
XX= 122.5894 YX= 7.7244 ZX= 12.3419
XY= 0.9042 YY= 107.8566 ZY= -13.6087
XZ= 11.8320 YZ= -5.8720 ZZ= 114.6329
Eigenvalues: 96.3411 117.1503 131.5875
21 O Isotropic = -31.2560 Anisotropy = 567.2190
XX= 65.3756 YX= 216.5989 ZX= -154.7336
XY= 183.7761 YY= 26.1918 ZY= -141.4158
XZ= -206.5636 YZ= -146.6200 ZZ= -185.3353
Eigenvalues: -286.6123 -154.0456 346.8900
22 C Isotropic = 181.7310 Anisotropy = 16.7729
XX= 184.0981 YX= -8.8261 ZX= -2.8921
XY= -10.1803 YY= 181.1498 ZY= 2.4376
XZ= -4.7856 YZ= -2.4339 ZZ= 179.9450
Eigenvalues: 172.1605 180.1196 192.9129
23 O Isotropic = 310.5067 Anisotropy = 76.9418
XX= 283.3327 YX= -15.7362 ZX= -4.8592
XY= -16.0262 YY= 328.5821 ZY= 42.2528
XZ= 6.3064 YZ= 29.1987 ZZ= 319.6053
Eigenvalues: 273.4716 296.2473 361.8012
24 C Isotropic = 129.6556 Anisotropy = 41.8397
XX= 152.1438 YX= -15.3226 ZX= -5.8476
XY= -12.3314 YY= 121.8397 ZY= -3.3922
XZ= 3.0362 YZ= -11.3362 ZZ= 114.9832
Eigenvalues: 108.2985 123.1195 157.5487
25 O Isotropic = 316.3618 Anisotropy = 57.3266
XX= 288.3031 YX= 12.2969 ZX= -14.7501
XY= -17.0231 YY= 308.3854 ZY= -5.4280
XZ= -4.7890 YZ= -6.9711 ZZ= 352.3970
Eigenvalues: 286.3562 308.1498 354.5795
26 H Isotropic = 27.4334 Anisotropy = 7.3682
XX= 25.7796 YX= -0.3148 ZX= 0.2437
XY= -0.6956 YY= 26.6791 ZY= -3.4488
XZ= -0.1784 YZ= -4.0536 ZZ= 29.8415
Eigenvalues: 24.0926 25.8621 32.3455
27 H Isotropic = 28.8939 Anisotropy = 6.1716
XX= 29.1306 YX= -0.0038 ZX= 4.3042
XY= 0.9499 YY= 30.8547 ZY= -1.7789
XZ= 3.5890 YZ= -3.5705 ZZ= 26.6965
Eigenvalues: 23.0129 30.6606 33.0083
28 H Isotropic = 30.3476 Anisotropy = 9.9624
XX= 28.8123 YX= 3.8975 ZX= -3.2652
XY= 2.6039 YY= 30.9417 ZY= -3.5894
XZ= -1.9654 YZ= -3.9498 ZZ= 31.2889
Eigenvalues: 26.4513 27.6023 36.9892
29 H Isotropic = 29.9486 Anisotropy = 9.5901
XX= 27.7513 YX= -1.2495 ZX= -1.0725
XY= -1.6293 YY= 34.3669 ZY= 3.3444
XZ= -1.4002 YZ= 3.8906 ZZ= 27.7274
Eigenvalues: 25.9324 27.5713 36.3420
30 H Isotropic = 30.9261 Anisotropy = 8.0386
XX= 31.8372 YX= -2.3219 ZX= 3.8474
XY= -2.6429 YY= 31.3746 ZY= -1.9534
XZ= 3.3799 YZ= -1.3166 ZZ= 29.5666
Eigenvalues: 26.9072 29.5860 36.2852
31 H Isotropic = 30.1069 Anisotropy = 9.6809
XX= 30.9919 YX= 0.2389 ZX= -5.7798
XY= -0.3324 YY= 29.4270 ZY= 2.0198
XZ= -6.0565 YZ= 1.1292 ZZ= 29.9018
Eigenvalues: 24.2513 29.5085 36.5608
32 H Isotropic = 30.6848 Anisotropy = 8.7177
XX= 31.1493 YX= 1.8459 ZX= -0.7625
XY= 2.0513 YY= 35.2743 ZY= 2.8905
XZ= -1.7351 YZ= 2.6745 ZZ= 25.6310
Eigenvalues: 24.4359 31.1220 36.4967
33 H Isotropic = 29.8454 Anisotropy = 10.7684
XX= 32.4039 YX= 0.0007 ZX= 5.1889
XY= -0.6988 YY= 25.5774 ZY= 0.6309
XZ= 4.8264 YZ= 1.5151 ZZ= 31.5547
Eigenvalues: 25.0156 27.4962 37.0243
34 H Isotropic = 29.1431 Anisotropy = 8.6641
XX= 29.6090 YX= 1.5200 ZX= 0.5830
XY= 1.2671 YY= 34.1934 ZY= 2.2366

XZ= -0.9933 YZ= 1.9026 ZZ= 23.6270
Eigenvalues: 23.2023 29.3080 34.9192
35 H Isotropic = 28.6668 Anisotropy = 11.0013
XX= 35.3604 YX= 0.4709 ZX= -2.5937
XY= 2.4749 YY= 26.1269 ZY= 0.6550
XZ= -2.0155 YZ= 0.8325 ZZ= 24.5131
Eigenvalues: 23.6052 26.3942 36.0010
36 H Isotropic = 29.2382 Anisotropy = 6.6608
XX= 27.3187 YX= 0.3815 ZX= -1.5990
XY= 0.6817 YY= 29.8787 ZY= -3.8012
XZ= -2.3481 YZ= -2.0570 ZZ= 30.5171
Eigenvalues: 26.1047 27.9312 33.6787
37 H Isotropic = 27.7838 Anisotropy = 10.7950
XX= 26.6089 YX= -2.0140 ZX= -1.2800
XY= -3.5353 YY= 29.3818 ZY= 5.4737
XZ= -1.3934 YZ= 5.4139 ZZ= 27.3606
Eigenvalues: 22.6581 25.7128 34.9805
38 H Isotropic = 28.3588 Anisotropy = 7.1180
XX= 26.3177 YX= -0.6284 ZX= 0.1297
XY= 1.4363 YY= 25.7015 ZY= 0.2816
XZ= -1.2076 YZ= 0.1336 ZZ= 33.0571
Eigenvalues: 25.4772 26.4950 33.1041
39 H Isotropic = 29.3353 Anisotropy = 5.3905
XX= 31.2633 YX= -1.1368 ZX= -0.7073
XY= -1.7580 YY= 31.2920 ZY= 0.6532
XZ= -0.6731 YZ= 1.4499 ZZ= 25.4505
Eigenvalues: 25.2340 29.8428 32.9289
40 H Isotropic = 29.9509 Anisotropy = 5.2377
XX= 28.9253 YX= 1.3837 ZX= 0.9945
XY= 1.8029 YY= 29.1044 ZY= -2.2209
XZ= 1.3953 YZ= -3.0595 ZZ= 31.8230
Eigenvalues: 26.1004 30.3096 33.4427
41 H Isotropic = 27.7744 Anisotropy = 7.1141
XX= 27.1279 YX= -1.0169 ZX= -0.0646
XY= -1.4137 YY= 26.8910 ZY= -5.2446
XZ= -0.0446 YZ= -3.0727 ZZ= 29.3045
Eigenvalues: 23.4864 27.3198 32.5172
42 H Isotropic = 26.2155 Anisotropy = 6.0199
XX= 26.2019 YX= -0.2481 ZX= -0.5242
XY= 0.6433 YY= 30.2033 ZY= 0.8361
XZ= -0.3880 YZ= -1.4971 ZZ= 22.2413
Eigenvalues: 22.1777 26.2400 30.2287
43 H Isotropic = 28.0530 Anisotropy = 3.6097
XX= 29.1463 YX= -0.9105 ZX= 0.7021
XY= -0.8383 YY= 25.1233 ZY= -1.7089
XZ= -0.3766 YZ= -1.3081 ZZ= 29.8893
Eigenvalues: 24.5479 29.1516 30.4594
44 H Isotropic = 28.4317 Anisotropy = 6.3118
XX= 30.1855 YX= -1.9573 ZX= -1.8461
XY= 0.3504 YY= 31.9469 ZY= -2.4366
XZ= -2.1894 YZ= -2.5500 ZZ= 23.1627
Eigenvalues: 21.9402 30.7154 32.6396
45 H Isotropic = 26.9489 Anisotropy = 3.9481
XX= 26.3948 YX= -0.6868 ZX= 0.1376
XY= -1.0607 YY= 24.9833 ZY= -1.2505
XZ= -0.4406 YZ= -0.1868 ZZ= 29.4685
Eigenvalues: 24.4638 26.8019 29.5809
46 H Isotropic = 29.9040 Anisotropy = 5.8897
XX= 31.9068 YX= -0.8021 ZX= 2.9426
XY= -1.3709 YY= 28.6462 ZY= 0.1058
XZ= 2.1297 YZ= -2.1680 ZZ= 29.1590
Eigenvalues: 27.5084 28.3731 33.8305
47 H Isotropic = 25.1042 Anisotropy = 4.6665
XX= 25.0567 YX= -2.0560 ZX= -2.8159
XY= -1.0547 YY= 23.9301 ZY= 0.3886
XZ= 0.7014 YZ= 3.2193 ZZ= 26.3260
Eigenvalues: 22.5643 24.5331 28.2153
48 H Isotropic = 25.5820 Anisotropy = 8.3078
XX= 24.0931 YX= 3.1933 ZX= -0.6931
XY= 2.8594 YY= 29.4470 ZY= -1.1690
XZ= -0.3986 YZ= -1.7756 ZZ= 23.2059
Eigenvalues: 22.6788 22.9467 31.1206
49 H Isotropic = 28.5747 Anisotropy = 20.6837
XX= 23.7038 YX= -4.8907 ZX= 1.3595

XY= -5.5497 YY= 33.3387 ZY= 10.4216
XZ= 0.0967 YZ= 10.3119 ZZ= 28.6816
Eigenvalues: 17.6975 25.6628 42.3638
50 H Isotropic = 29.6811 Anisotropy = 14.3756
XX= 31.0769 YX= -2.5160 ZX= 1.9452
XY= -0.1595 YY= 19.0805 ZY= -0.7469
XZ= 1.5154 YZ= 0.2861 ZZ= 38.8860
Eigenvalues: 18.9331 30.8455 39.2649

Conformer 3

1 C Isotropic = 118.5093 Anisotropy = 34.9059
XX= 129.2366 YX= -4.4025 ZX= 8.7330
XY= -8.0950 YY= 120.8610 ZY= -16.6286
XZ= 12.7676 YZ= -16.6233 ZZ= 105.4304
Eigenvalues: 93.8077 119.9403 141.7799
2 C Isotropic = 160.1555 Anisotropy = 29.6971
XX= 171.8442 YX= 13.5787 ZX= -11.1326
XY= 9.2688 YY= 154.8197 ZY= 5.6313
XZ= -13.8014 YZ= 13.6007 ZZ= 153.8025
Eigenvalues: 136.5761 163.9368 179.9535
3 C Isotropic = 143.1046 Anisotropy = 60.7768
XX= 131.1623 YX= 26.3192 ZX= 4.7257
XY= 26.3719 YY= 169.6561 ZY= 3.7847
XZ= -3.6487 YZ= 8.4095 ZZ= 128.4953
Eigenvalues: 117.2963 128.3950 183.6225
4 N Isotropic = 209.0476 Anisotropy = 68.5063
XX= 210.2106 YX= 55.4629 ZX= -31.7658
XY= 48.7709 YY= 188.9686 ZY= 32.4824
XZ= -36.5816 YZ= 44.4280 ZZ= 227.9638
Eigenvalues: 121.5623 250.8621 254.7185
5 C Isotropic = 116.8525 Anisotropy = 44.2724
XX= 138.0886 YX= 4.7984 ZX= -18.3543
XY= 12.5998 YY= 107.0901 ZY= 14.9947
XZ= -17.6986 YZ= 8.4153 ZZ= 105.3789
Eigenvalues: 87.1537 117.0365 146.3675
6 C Isotropic = 133.0753 Anisotropy = 54.1192
XX= 127.2099 YX= 16.7443 ZX= 20.0902
XY= 19.1617 YY= 125.8010 ZY= 11.7335
XZ= 18.3653 YZ= 16.7005 ZZ= 146.2151
Eigenvalues: 108.1197 121.9515 169.1548
7 C Isotropic = 55.7005 Anisotropy = 123.8442
XX= -4.5340 YX= -61.8636 ZX= -9.3819
XY= -62.3788 YY= 94.1115 ZY= -47.5873
XZ= 10.0495 YZ= -16.6291 ZZ= 77.5240
Eigenvalues: -36.2519 65.0901 138.2633
8 C Isotropic = 65.8620 Anisotropy = 125.3808
XX= 8.5863 YX= -65.6005 ZX= 3.3645
XY= -76.6048 YY= 109.3007 ZY= -10.8823
XZ= -20.3851 YZ= -32.0505 ZZ= 79.6990
Eigenvalues: -30.9602 79.0970 149.4492
9 O Isotropic = 129.4930 Anisotropy = 128.5389
XX= 128.9665 YX= 37.4143 ZX= -90.8094
XY= -35.3546 YY= 215.0328 ZY= -53.5212
XZ= -142.5405 YZ= 58.9929 ZZ= 44.4797
Eigenvalues: -37.3955 210.6889 215.1856
10 C Isotropic = 32.8522 Anisotropy = 81.0290
XX= 49.7647 YX= 43.8273 ZX= -28.5726
XY= 65.3831 YY= -16.5379 ZY= 28.7893
XZ= -14.1141 YZ= 4.7964 ZZ= 65.3297
Eigenvalues: -52.6451 64.3301 86.8715
11 C Isotropic = 63.5951 Anisotropy = 119.5365
XX= 31.8504 YX= -24.2747 ZX= -79.9763
XY= -25.0275 YY= 79.9016 ZY= 4.6690
XZ= -73.5768 YZ= 12.6598 ZZ= 79.0333
Eigenvalues: -26.9493 74.4485 143.2861
12 O Isotropic = -79.2794 Anisotropy = 584.3785
XX= 86.5086 YX= 91.2297 ZX= -230.5198
XY= 98.4667 YY= -215.3394 ZY= -131.7104
XZ= -266.4159 YZ= -185.4716 ZZ= -109.0075
Eigenvalues: -348.1783 -199.9662 310.3062
13 C Isotropic = 57.8176 Anisotropy = 147.1143
XX= 29.5453 YX= -17.6008 ZX= -93.3792
XY= -16.1548 YY= 67.5586 ZY= 26.0298
XZ= -89.1976 YZ= 17.9817 ZZ= 76.3489

Eigenvalues: -41.2933 58.8523 155.8938
14 C Isotropic = 178.7625 Anisotropy = 22.8827
XX= 186.5798 YX= 13.5839 ZX= -3.9263
XY= 9.7216 YY= 175.7568 ZY= 7.8370
XZ= -4.0677 YZ= 5.2506 ZZ= 173.9509
Eigenvalues: 162.9684 179.3015 194.0176
15 C Isotropic = 156.8566 Anisotropy = 20.8567
XX= 165.8326 YX= 8.6952 ZX= -1.3608
XY= 8.3020 YY= 155.8182 ZY= -0.8564
XZ= -1.5081 YZ= 0.8351 ZZ= 148.9191
Eigenvalues: 148.6359 151.1729 170.7611
16 C Isotropic = 157.4223 Anisotropy = 20.6423
XX= 162.6060 YX= 8.7894 ZX= -1.0555
XY= 9.7146 YY= 161.1838 ZY= 3.7987
XZ= 1.6688 YZ= -5.8352 ZZ= 148.4771
Eigenvalues: 148.2581 152.8250 171.1838
17 C Isotropic = 133.4183 Anisotropy = 54.9097
XX= 145.1181 YX= -24.9435 ZX= -11.9483
XY= -21.7200 YY= 147.9768 ZY= -11.6639
XZ= -6.9134 YZ= -12.7452 ZZ= 107.1601
Eigenvalues: 97.8987 132.3315 170.0248
18 O Isotropic = 143.7082 Anisotropy = 148.7889
XX= 188.8175 YX= 57.5370 ZX= -27.5415
XY= 89.2714 YY= 93.0193 ZY= -45.8932
XZ= 67.1941 YZ= -139.6701 ZZ= 149.2878
Eigenvalues: -2.4696 190.6934 242.9008
19 C Isotropic = 19.6907 Anisotropy = 96.5227
XX= 9.4693 YX= 60.2394 ZX= -41.3498
XY= 43.5176 YY= 44.0808 ZY= 19.8837
XZ= -65.4334 YZ= 29.4443 ZZ= 5.5219
Eigenvalues: -71.6010 46.6338 84.0391
20 C Isotropic = 114.8746 Anisotropy = 24.7893
XX= 119.3848 YX= 9.3086 ZX= 11.9457
XY= 4.9495 YY= 109.2732 ZY= -11.3110
XZ= 14.6047 YZ= -11.8599 ZZ= 115.9656
Eigenvalues: 93.0876 120.1353 131.4008
21 O Isotropic = -40.4290 Anisotropy = 573.4709
XX= 68.8838 YX= 226.4479 ZX= -143.9067
XY= 199.6592 YY= 33.3054 ZY= -125.2422
XZ= -187.7451 YZ= -130.2173 ZZ= -223.4762
Eigenvalues: -302.4953 -160.6766 341.8849
22 C Isotropic = 183.7290 Anisotropy = 19.9612
XX= 185.7622 YX= -10.6927 ZX= -3.6966
XY= -12.9452 YY= 183.6566 ZY= 1.5162
XZ= -3.4157 YZ= -1.4872 ZZ= 181.7683
Eigenvalues: 172.2267 181.9238 197.0365
23 O Isotropic = 305.2867 Anisotropy = 58.6318
XX= 290.8429 YX= -30.6002 ZX= -5.4134
XY= -27.6702 YY= 300.9601 ZY= 17.2013
XZ= 0.1807 YZ= 27.1258 ZZ= 324.0571
Eigenvalues: 263.5872 307.8983 344.3746
24 C Isotropic = 130.7371 Anisotropy = 59.8579
XX= 122.5445 YX= 7.7065 ZX= 10.8417
XY= 6.2953 YY= 102.7784 ZY= -6.1348
XZ= 15.7687 YZ= -2.1674 ZZ= 166.8885
Eigenvalues: 99.5234 122.0456 170.6424
25 O Isotropic = 310.7733 Anisotropy = 48.5078
XX= 334.8131 YX= -17.4573 ZX= 0.9961
XY= -20.1926 YY= 298.6459 ZY= 0.8276
XZ= -10.0305 YZ= -4.1534 ZZ= 298.8608
Eigenvalues: 289.4408 299.7672 343.1118
26 H Isotropic = 27.4402 Anisotropy = 7.3399
XX= 25.9120 YX= -0.2051 ZX= 0.1538
XY= -0.5716 YY= 26.7510 ZY= -3.5336
XZ= -0.3156 YZ= -4.1884 ZZ= 29.6575
Eigenvalues: 24.0086 25.9785 32.3334
27 H Isotropic = 28.9209 Anisotropy = 6.1196
XX= 29.0482 YX= 0.1771 ZX= 4.2155
XY= 1.0890 YY= 30.7679 ZY= -1.9650
XZ= 3.4701 YZ= -3.7113 ZZ= 26.9465
Eigenvalues: 23.0860 30.6760 33.0006
28 H Isotropic = 30.3408 Anisotropy = 10.0018
XX= 28.9758 YX= 3.8323 ZX= -3.5217
XY= 2.6046 YY= 30.6151 ZY= -3.4861

XZ= -2.2060 YZ= -3.8636 ZZ= 31.4316
Eigenvalues: 26.4742 27.5396 37.0087
29 H Isotropic = 29.9337 Anisotropy = 9.6442
XX= 27.6504 YX= -1.1983 ZX= -0.9764
XY= -1.6072 YY= 34.6746 ZY= 3.1216
XZ= -1.2777 YZ= 3.6669 ZZ= 27.4761
Eigenvalues: 25.9313 27.5066 36.3632
30 H Isotropic = 30.8608 Anisotropy = 9.3675
XX= 32.6842 YX= -2.9562 ZX= 4.2804
XY= -3.5104 YY= 30.6032 ZY= -1.1985
XZ= 3.8815 YZ= -1.0149 ZZ= 29.2949
Eigenvalues: 26.3223 29.1543 37.1058
31 H Isotropic = 30.5179 Anisotropy = 10.1839
XX= 29.6653 YX= -1.4837 ZX= -5.8298
XY= -2.1345 YY= 29.7881 ZY= 1.3275
XZ= -5.8148 YZ= 0.5919 ZZ= 32.1002
Eigenvalues: 24.7986 29.4479 37.3071
32 H Isotropic = 30.7164 Anisotropy = 8.6193
XX= 30.8655 YX= 1.4716 ZX= -1.3344
XY= 1.7803 YY= 35.8192 ZY= 1.9307
XZ= -2.0720 YZ= 1.7368 ZZ= 25.4644
Eigenvalues: 24.5319 31.1546 36.4626
33 H Isotropic = 29.8520 Anisotropy = 10.7327
XX= 32.0406 YX= -0.0880 ZX= 5.1471
XY= -0.7411 YY= 25.5585 ZY= 0.4779
XZ= 4.8469 YZ= 1.3752 ZZ= 31.9569
Eigenvalues: 25.0786 27.4702 37.0072
34 H Isotropic = 29.0064 Anisotropy = 8.7025
XX= 29.5318 YX= 1.3966 ZX= 0.8983
XY= 1.1169 YY= 34.0590 ZY= 2.3880
XZ= -0.7523 YZ= 2.1016 ZZ= 23.4284
Eigenvalues: 22.9689 29.2422 34.8081
35 H Isotropic = 28.6357 Anisotropy = 11.0523
XX= 35.5396 YX= 0.4475 ZX= -2.1775
XY= 2.3994 YY= 26.0498 ZY= 0.7580
XZ= -1.5341 YZ= 0.8528 ZZ= 24.3176
Eigenvalues: 23.5733 26.3298 36.0038
36 H Isotropic = 29.2250 Anisotropy = 6.7589
XX= 27.4522 YX= 0.5143 ZX= -1.7705
XY= 0.8503 YY= 29.9534 ZY= -3.8047
XZ= -2.5549 YZ= -2.0690 ZZ= 30.2692
Eigenvalues: 26.0116 27.9324 33.7309
37 H Isotropic = 27.7726 Anisotropy = 10.8170
XX= 26.6824 YX= -2.1205 ZX= -1.4376
XY= -3.6230 YY= 28.9977 ZY= 5.4045
XZ= -1.6171 YZ= 5.4173 ZZ= 27.6378
Eigenvalues: 22.6806 25.6533 34.9839
38 H Isotropic = 28.3323 Anisotropy = 7.0634
XX= 26.2630 YX= -0.7228 ZX= -0.0142
XY= 1.3679 YY= 25.7612 ZY= 0.0974
XZ= -1.3468 YZ= -0.1089 ZZ= 32.9727
Eigenvalues: 25.5910 26.3647 33.0412
39 H Isotropic = 29.4173 Anisotropy = 5.4157
XX= 30.7813 YX= -1.0393 ZX= -1.0645
XY= -1.7808 YY= 31.6654 ZY= 0.7756
XZ= -1.0196 YZ= 1.5033 ZZ= 25.8051
Eigenvalues: 25.4727 29.7514 33.0277
40 H Isotropic = 29.9794 Anisotropy = 5.7559
XX= 28.8565 YX= 1.0971 ZX= 1.3498
XY= 1.4313 YY= 29.1890 ZY= -2.5633
XZ= 1.2531 YZ= -3.3206 ZZ= 31.8927
Eigenvalues: 26.1433 29.9782 33.8167
41 H Isotropic = 27.7304 Anisotropy = 7.1683
XX= 27.1453 YX= -1.2955 ZX= -0.3054
XY= -1.6560 YY= 27.4572 ZY= -5.4668
XZ= -0.2934 YZ= -3.2156 ZZ= 28.5887
Eigenvalues: 23.2051 27.4769 32.5093
42 H Isotropic = 26.1051 Anisotropy = 6.2071
XX= 25.9601 YX= -0.2714 ZX= -0.6041
XY= 0.5054 YY= 30.2339 ZY= 1.3900
XZ= -0.5450 YZ= -0.9169 ZZ= 22.1212
Eigenvalues: 22.0294 26.0427 30.2432
43 H Isotropic = 28.4195 Anisotropy = 6.4154
XX= 28.9546 YX= 4.2944 ZX= 0.1274

XY= 3.9961 YY= 27.9287 ZY= -0.6625
XZ= 0.4560 YZ= 1.7532 ZZ= 28.3750
Eigenvalues: 24.2534 28.3085 32.6964
44 H Isotropic = 27.9959 Anisotropy = 5.1638
XX= 31.2708 YX= -1.3816 ZX= -0.8144
XY= -0.5991 YY= 25.5641 ZY= -0.1004
XZ= 0.4232 YZ= -1.5681 ZZ= 27.1528
Eigenvalues: 25.0494 27.4999 31.4385
45 H Isotropic = 26.9840 Anisotropy = 3.8092
XX= 26.7496 YX= -0.6136 ZX= -0.0578
XY= -0.8758 YY= 24.8566 ZY= -1.4373
XZ= -0.5816 YZ= -0.3208 ZZ= 29.3457
Eigenvalues: 24.4211 27.0074 29.5234
46 H Isotropic = 29.6854 Anisotropy = 4.9401
XX= 31.8669 YX= -0.0716 ZX= 1.9598
XY= -0.5639 YY= 28.4304 ZY= -1.0236
XZ= 1.5373 YZ= -3.0969 ZZ= 28.7588
Eigenvalues: 26.3462 29.7311 32.9788
47 H Isotropic = 25.0678 Anisotropy = 4.6752
XX= 24.9794 YX= -2.0335 ZX= -2.8817
XY= -1.1390 YY= 23.7949 ZY= 0.1424
XZ= 0.5566 YZ= 3.0922 ZZ= 26.4292
Eigenvalues: 22.5407 24.4782 28.1846
48 H Isotropic = 25.6002 Anisotropy = 8.1491
XX= 24.0963 YX= 3.0523 ZX= -0.6869
XY= 2.8082 YY= 29.3532 ZY= -1.3249
XZ= -0.4360 YZ= -1.8743 ZZ= 23.3510
Eigenvalues: 22.7176 23.0501 31.0329
49 H Isotropic = 28.8205 Anisotropy = 19.4943
XX= 24.4779 YX= -5.5429 ZX= 0.2265
XY= -5.6744 YY= 30.7341 ZY= 9.3912
XZ= -0.7828 YZ= 10.2177 ZZ= 31.2495
Eigenvalues: 18.4243 26.2205 41.8167
50 H Isotropic = 30.5255 Anisotropy = 18.1373
XX= 36.2486 YX= -10.4793 ZX= -0.5029
XY= -9.4366 YY= 26.6856 ZY= -2.6358
XZ= 0.8358 YZ= -1.3345 ZZ= 28.6425
Eigenvalues: 20.1211 28.8385 42.6171

Structure Coordinates of Caulamidine A from DFT Geometry Optimization

C	4.021	-1.026	-1.527
C	2.729	-0.675	-1.109
C	2.528	-0.27	0.227
C	3.604	-0.21	1.11
C	4.88	-0.556	0.667
C	5.099	-0.967	-0.648
N	1.679	-0.761	-2.029
C	0.556	-0.161	-1.783
C	1.11	0.02	0.638
C	-1.199	0.796	-0.128
C	0.313	0.675	-0.515
C	-1.377	1.864	0.964
N	-0.817	3.101	0.798
C	0.567	3.135	0.291
C	0.849	2.113	-0.814
N	-2.105	1.506	1.978
C	-1.108	4.099	1.823
Cl	6.236	-0.471	1.789
C	-2.429	0.151	1.763
C	-1.864	-0.372	0.58
C	-2.005	-1.713	0.255
C	-2.747	-2.522	1.127
C	-3.33	-2.012	2.286
C	-3.17	-0.662	2.615
Cl	-2.94	-4.231	0.742
C	-1.967	1.212	-1.41
N	-0.409	-0.151	-2.764
C	-1.811	0.17	-2.509
C	-0.158	-0.89	-3.999
Cl	-3.745	1.483	-1.142

H	-1.604	2.181	-1.747
H	-2.173	4.074	2.055
H	-0.837	5.086	1.441
H	-0.552	3.908	2.752
H	0.895	-0.803	-4.259
H	-0.78	-0.469	-4.794
H	-0.394	-1.957	-3.892
H	0.617	-0.921	0.908
H	1.074	0.651	1.533
H	0.761	4.141	-0.094
H	1.261	2.982	1.133
H	0.436	2.469	-1.761
H	1.933	2.059	-0.955
H	-2.243	0.565	-3.433
H	-2.387	-0.728	-2.245
H	4.162	-1.342	-2.556
H	3.453	0.098	2.14
H	6.097	-1.234	-0.976
H	-1.55	-2.151	-0.627
H	-3.899	-2.671	2.933
H	-3.605	-0.254	3.52

GIAO Chemical Shielding Tensors of Caulamidine A

```
    1  C    Isotropic =    73.3703   Anisotropy =   141.2073
  XX=    61.7645   YX=    20.1256   ZX=     8.3978
  XY=    22.6134   YY=   150.8281   ZY=   -45.4062
  XZ=     4.3523   YZ=   -46.0083   ZZ=     7.5184
  Eigenvalues:    -7.9826    60.5851   167.5086
    2  C    Isotropic =    56.1240   Anisotropy =   151.0068
  XX=     2.2535   YX=    21.9017   ZX=   -31.7615
  XY=     5.5815   YY=   147.1450   ZY=   -30.3660
  XZ=   -38.4038   YZ=   -29.5607   ZZ=    18.9735
  Eigenvalues:   -25.8332    37.4100   156.7952
    3  C    Isotropic =    73.9681   Anisotropy =   155.5631
  XX=    11.4624   YX=    28.4309   ZX=    14.6050
  XY=    30.6791   YY=   164.5843   ZY=   -31.0167
  XZ=     7.3042   YZ=   -36.9867   ZZ=    45.8575
  Eigenvalues:    -1.0643    45.2916   177.6768
    4  C    Isotropic =    72.1485   Anisotropy =   124.9639
  XX=    71.4064   YX=    14.7081   ZX=     6.0047
  XY=     8.8009   YY=   141.5472   ZY=   -44.4196
  XZ=    12.3490   YZ=   -44.2133   ZZ=     3.4921
  Eigenvalues:   -11.2938    72.2816   155.4578
    5  C    Isotropic =    62.7872   Anisotropy =    96.2986
  XX=    23.5580   YX=     4.1230   ZX=   -47.1358
  XY=     5.5475   YY=   122.6791   ZY=   -11.9696
  XZ=   -47.2617   YZ=   -15.7686   ZZ=    42.1245
  Eigenvalues:   -15.4510    76.8263   126.9863
    6  C    Isotropic =    71.0978   Anisotropy =   141.3478
  XX=     1.3397   YX=    33.9428   ZX=    19.4546
  XY=    34.0807   YY=   150.8592   ZY=   -30.3426
  XZ=    16.5239   YZ=   -32.1980   ZZ=    61.0945
  Eigenvalues:   -14.2618    62.2255   165.3297
    7  N    Isotropic =    30.5974   Anisotropy =   329.5536
  XX=   -90.3982   YX=    72.9496   ZX=  -107.6496
  XY=   101.8368   YY=   176.1129   ZY=   -78.8872
  XZ=  -107.6878   YZ=   -74.5075   ZZ=     6.0775
  Eigenvalues:  -163.6262     5.1186   250.2998
    8  C    Isotropic =    42.1054   Anisotropy =   122.4684
  XX=    36.7657   YX=    10.4281   ZX=   -55.4613
  XY=    38.1858   YY=    90.2246   ZY=   -33.8850
  XZ=   -19.9424   YZ=   -52.5531   ZZ=    -0.6742
  Eigenvalues:   -29.8249    32.3900   123.7510
    9  C    Isotropic =   163.9295   Anisotropy =    30.7497
  XX=   183.1890   YX=    -9.7276   ZX=    -2.1628
  XY=    -2.3796   YY=   149.9786   ZY=    -7.5451
  XZ=     4.2144   YZ=    -4.5715   ZZ=   158.6209
  Eigenvalues:   146.1904   161.1687   184.4293
   10  C    Isotropic =   133.6350   Anisotropy =     8.4992
  XX=   132.1560   YX=     0.4894   ZX=     2.3603
  XY=     2.4525   YY=   138.5751   ZY=    -3.1071
```

XZ= -0.3945 YZ= -1.1990 ZZ= 130.1738
Eigenvalues: 129.0778 132.5260 139.3011
11 C Isotropic = 149.8353 Anisotropy = 10.5135
XX= 150.8640 YX= 6.5660 ZX= -1.5064
XY= 0.6875 YY= 154.3546 ZY= 5.0037
XZ= -4.6389 YZ= 2.2930 ZZ= 144.2872
Eigenvalues: 141.3115 151.3500 156.8443
12 C Isotropic = 25.6525 Anisotropy = 119.8213
XX= 61.0411 YX= -45.6530 ZX= 34.0583
XY= -22.0725 YY= -24.2707 ZY= -58.7191
XZ= 29.5146 YZ= -26.0970 ZZ= 40.1869
Eigenvalues: -48.1664 19.5904 105.5333
13 N Isotropic = 178.6561 Anisotropy = 57.0590
XX= 179.2225 YX= 16.6399 ZX= 34.0686
XY= -3.7729 YY= 206.5919 ZY= 8.9946
XZ= 54.6386 YZ= -1.7402 ZZ= 150.1540
Eigenvalues: 118.0060 201.2669 216.6954
14 C Isotropic = 146.4623 Anisotropy = 52.3242
XX= 172.5768 YX= 3.0525 ZX= -21.9418
XY= 1.9261 YY= 131.6762 ZY= 9.6545
XZ= -18.1439 YZ= 10.5424 ZZ= 135.1341
Eigenvalues: 118.4499 139.5919 181.3452
15 C Isotropic = 168.0703 Anisotropy = 18.5335
XX= 154.2974 YX= 10.3105 ZX= -8.0709
XY= -4.0269 YY= 177.7419 ZY= 2.0173
XZ= 1.3805 YZ= 7.2840 ZZ= 172.1715
Eigenvalues: 153.0483 170.7366 180.4259
16 N Isotropic = 1.8926 Anisotropy = 324.9541
XX= 109.3593 YX= -142.5246 ZX= 88.8207
XY= -130.9505 YY= -145.5634 ZY= -10.5896
XZ= 118.5731 YZ= 24.8457 ZZ= 41.8819
Eigenvalues: -214.8154 1.9646 218.5287
17 C Isotropic = 157.1465 Anisotropy = 53.7879
XX= 142.9931 YX= -8.8105 ZX= -6.3505
XY= -14.4810 YY= 166.4434 ZY= 25.7413
XZ= -9.1196 YZ= 23.9804 ZZ= 162.0031
Eigenvalues: 137.1422 141.2923 193.0051
18 Cl Isotropic = 745.6722 Anisotropy = 445.7861
XX= 857.7816 YX= 20.1521 ZX= 222.5571
XY= 17.9074 YY= 606.9492 ZY= 11.0637
XZ= 222.9181 YZ= 9.2948 ZZ= 772.2857
Eigenvalues: 587.2543 606.8993 1042.8629
19 C Isotropic = 42.6006 Anisotropy = 133.3178
XX= 104.8531 YX= -19.7264 ZX= 32.8428
XY= -32.9676 YY= -27.9125 ZY= -24.0419
XZ= 38.3681 YZ= -38.1621 ZZ= 50.8613
Eigenvalues: -40.0820 36.4047 131.4792
20 C Isotropic = 64.8497 Anisotropy = 140.7011
XX= 109.2979 YX= -33.8811 ZX= 70.7649
XY= -33.1744 YY= 27.3681 ZY= 4.6508
XZ= 60.6524 YZ= 8.7023 ZZ= 57.8830
Eigenvalues: -6.5843 42.4829 158.6504
21 C Isotropic = 74.9504 Anisotropy = 134.3850
XX= 124.2024 YX= 0.8888 ZX= 63.4456
XY= -5.8062 YY= 55.9753 ZY= -31.4389
XZ= 68.0744 YZ= -34.6500 ZZ= 44.6735
Eigenvalues: -5.2541 65.5649 164.5404
22 C Isotropic = 63.0850 Anisotropy = 95.0787
XX= 110.5856 YX= -21.6542 ZX= 17.3036
XY= -19.1233 YY= -7.8558 ZY= -23.7352
XZ= 18.1375 YZ= -24.2073 ZZ= 86.5251
Eigenvalues: -15.6206 78.4047 126.4708
23 C Isotropic = 69.0081 Anisotropy = 145.8769
XX= 111.3271 YX= -44.5049 ZX= 70.7496
XY= -41.9390 YY= 36.3844 ZY= 12.9909
XZ= 69.3082 YZ= 14.8894 ZZ= 59.3127
Eigenvalues: -16.3294 57.0944 166.2593
24 C Isotropic = 78.7263 Anisotropy = 147.2814
XX= 131.5354 YX= -12.2772 ZX= 69.1256
XY= -12.2632 YY= 56.5800 ZY= -33.2231
XZ= 70.0949 YZ= -30.7667 ZZ= 48.0635
Eigenvalues: -0.3500 59.6151 176.9139
25 Cl Isotropic = 744.3695 Anisotropy = 449.2518
XX= 606.3369 YX= 44.4931 ZX= 7.9014

XY= 46.1914 YY= 1017.6208 ZY= 97.2326
XZ= 16.9450 YZ= 93.6977 ZZ= 609.1508
Eigenvalues: 587.5620 601.6758 1043.8707
26 C Isotropic = 131.9597 Anisotropy = 37.9082
XX= 155.8470 YX= -3.7316 ZX= 0.6133
XY= 0.2230 YY= 103.6539 ZY= 1.3887
XZ= -10.9790 YZ= 2.7745 ZZ= 136.3782
Eigenvalues: 103.4827 135.1646 157.2318
27 N Isotropic = 168.4667 Anisotropy = 63.9112
XX= 146.8593 YX= 31.1782 ZX= 41.2037
XY= 27.8584 YY= 165.3508 ZY= -15.8643
XZ= 24.7464 YZ= -3.3920 ZZ= 193.1901
Eigenvalues: 112.0381 182.2880 211.0742
28 C Isotropic = 142.1216 Anisotropy = 55.2026
XX= 165.5857 YX= -16.7552 ZX= -11.1831
XY= -17.0915 YY= 126.2872 ZY= 10.2945
XZ= -17.2476 YZ= 14.0455 ZZ= 134.4918
Eigenvalues: 116.6099 130.8314 178.9233
29 C Isotropic = 158.5209 Anisotropy = 51.2351
XX= 139.0610 YX= -3.0297 ZX= -7.1055
XY= -10.0630 YY= 153.8083 ZY= 16.0405
XZ= -10.2752 YZ= 17.9747 ZZ= 182.6934
Eigenvalues: 136.4199 146.4652 192.6777
30 Cl Isotropic = 825.2891 Anisotropy = 398.7389
XX= 1067.8549 YX= -88.1460 ZX= -36.1264
XY= -66.6517 YY= 691.7209 ZY= 24.1388
XZ= -61.5745 YZ= 43.7720 ZZ= 716.2915
Eigenvalues: 663.8824 720.8699 1091.1150
31 H Isotropic = 26.9536 Anisotropy = 5.4980
XX= 29.8788 YX= 1.5543 ZX= 0.9342
XY= 1.6556 YY= 26.9053 ZY= -1.4178
XZ= -0.3150 YZ= -2.3721 ZZ= 24.0767
Eigenvalues: 22.9752 27.2667 30.6189
32 H Isotropic = 28.0650 Anisotropy = 10.4456
XX= 30.1133 YX= -2.0861 ZX= -3.6483
XY= -3.1952 YY= 28.3072 ZY= 4.2407
XZ= -4.1561 YZ= 2.9942 ZZ= 25.7745
Eigenvalues: 22.6684 26.4978 35.0287
33 H Isotropic = 29.5112 Anisotropy = 10.8451
XX= 25.0117 YX= -0.6675 ZX= -1.1310
XY= -0.0672 YY= 36.4224 ZY= 1.0397
XZ= -0.8780 YZ= 2.3251 ZZ= 27.0994
Eigenvalues: 24.5992 27.1930 36.7413
34 H Isotropic = 29.0133 Anisotropy = 10.5733
XX= 25.3539 YX= 0.2474 ZX= 1.7516
XY= -0.1575 YY= 27.5358 ZY= 4.0078
XZ= 2.4191 YZ= 3.1407 ZZ= 34.1504
Eigenvalues: 24.5705 26.4073 36.0622
35 H Isotropic = 27.2668 Anisotropy = 9.9417
XX= 30.0622 YX= -0.6155 ZX= -2.5279
XY= -1.5669 YY= 21.4167 ZY= 2.0695
XZ= -3.9125 YZ= 2.5936 ZZ= 30.3215
Eigenvalues: 20.8327 27.0731 33.8946
36 H Isotropic = 29.6406 Anisotropy = 10.3484
XX= 26.1726 YX= -1.4245 ZX= 0.7071
XY= -1.5115 YY= 26.4451 ZY= 0.7749
XZ= 2.3895 YZ= 0.0732 ZZ= 36.3041
Eigenvalues: 24.6564 27.7259 36.5395
37 H Isotropic = 28.8680 Anisotropy = 10.8856
XX= 24.2393 YX= 0.6753 ZX= -0.6464
XY= 1.2875 YY= 33.8310 ZY= 3.7728
XZ= -0.7149 YZ= 4.5161 ZZ= 28.5335
Eigenvalues: 23.7602 26.7186 36.1250
38 H Isotropic = 29.0276 Anisotropy = 10.6224
XX= 35.3426 YX= 1.4134 ZX= -2.1505
XY= 2.6187 YY= 28.0268 ZY= -4.2969
XZ= 0.6384 YZ= -3.1095 ZZ= 23.7134
Eigenvalues: 21.5754 29.3982 36.1092
39 H Isotropic = 29.7828 Anisotropy = 5.9403
XX= 31.8159 YX= -1.7767 ZX= 0.6611
XY= -3.0972 YY= 26.0882 ZY= 2.2371
XZ= 3.5257 YZ= 2.3867 ZZ= 31.4443
Eigenvalues: 24.0377 31.5678 33.7430
40 H Isotropic = 28.8647 Anisotropy = 9.0542

XX= 27.0462 YX= 4.0491 ZX= -2.9172
XY= 1.2815 YY= 33.8819 ZY= 0.1657
XZ= -2.2185 YZ= -0.3843 ZZ= 25.6660
Eigenvalues: 23.4511 28.2422 34.9009
41 H Isotropic = 28.7092 Anisotropy = 7.4608
XX= 32.0266 YX= 0.6688 ZX= 3.3941
XY= -1.2609 YY= 27.2452 ZY= 4.0218
XZ= 2.6607 YZ= 2.8103 ZZ= 26.8558
Eigenvalues: 22.9575 29.4871 33.6831
42 H Isotropic = 29.7416 Anisotropy = 6.1177
XX= 26.1179 YX= 2.4225 ZX= 0.0154
XY= -0.1327 YY= 30.5919 ZY= -2.0349
XZ= 2.1269 YZ= -2.0438 ZZ= 32.5151
Eigenvalues: 25.4854 29.9195 33.8201
43 H Isotropic = 29.9960 Anisotropy = 7.2667
XX= 33.9750 YX= 0.3413 ZX= -2.7971
XY= -2.6364 YY= 31.1269 ZY= 0.3502
XZ= -1.8709 YZ= -0.8745 ZZ= 24.8860
Eigenvalues: 24.2809 30.8665 34.8404
44 H Isotropic = 28.5233 Anisotropy = 7.6006
XX= 28.3106 YX= -3.4353 ZX= 3.0521
XY= -2.8245 YY= 24.3881 ZY= -0.0126
XZ= 0.6269 YZ= 0.6758 ZZ= 32.8711
Eigenvalues: 22.5173 29.4622 33.5904
45 H Isotropic = 28.0484 Anisotropy = 4.0128
XX= 28.0204 YX= 2.9271 ZX= 0.4015
XY= 2.3852 YY= 28.1132 ZY= 0.7291
XZ= -2.2452 YZ= 1.0154 ZZ= 28.0116
Eigenvalues: 24.8938 28.5278 30.7236
46 H Isotropic = 24.5374 Anisotropy = 6.5512
XX= 28.5921 YX= -1.7171 ZX= 0.3344
XY= -1.3646 YY= 21.0710 ZY= 1.2504
XZ= 0.6082 YZ= 1.3616 ZZ= 23.9492
Eigenvalues: 20.2567 24.4507 28.9049
47 H Isotropic = 24.9006 Anisotropy = 11.2089
XX= 31.6052 YX= -1.8748 ZX= 1.7725
XY= -2.0262 YY= 20.1046 ZY= 0.6602
XZ= 2.5401 YZ= 0.4247 ZZ= 22.9922
Eigenvalues: 19.5315 22.7972 32.3733
48 H Isotropic = 24.4840 Anisotropy = 5.9518
XX= 25.0908 YX= -0.6856 ZX= 1.2924
XY= -0.8746 YY= 20.7083 ZY= 2.1289
XZ= 0.7234 YZ= 2.2150 ZZ= 27.6530
Eigenvalues: 19.8796 25.1206 28.4519
49 H Isotropic = 24.7006 Anisotropy = 12.1858
XX= 21.6119 YX= 3.1805 ZX= -2.7905
XY= 5.1495 YY= 29.8404 ZY= -2.7189
XZ= -2.8492 YZ= -2.5623 ZZ= 22.6494
Eigenvalues: 18.9487 22.3286 32.8245
50 H Isotropic = 24.3207 Anisotropy = 6.0724
XX= 21.5565 YX= 0.4059 ZX= -2.9132
XY= 0.4218 YY= 25.8285 ZY= 2.2790
XZ= -3.0551 YZ= 1.9485 ZZ= 25.5772
Eigenvalues: 19.6509 24.9423 28.3690
51 H Isotropic = 24.2814 Anisotropy = 4.8745
XX= 22.2474 YX= 1.7701 ZX= -2.7518
XY= 1.9463 YY= 25.9261 ZY= -0.3684
XZ= -2.6241 YZ= -0.1247 ZZ= 24.6706
Eigenvalues: 20.1435 25.1695 27.5310

Structure Coordinates of the C26-inverted diastereomer of Caulamidine A from DFT Geometry Optimization

C	3.938	0.683	1.67
C	2.64	0.457	1.188
C	2.426	0.406	-0.205
C	3.495	0.567	-1.084
C	4.777	0.782	-0.578
C	5.009	0.843	0.796
Cl	6.124	0.978	-1.698
N	1.598	0.317	2.112
C	0.465	-0.187	1.732

C	1.003	0.233	-0.665
N	-0.495	-0.439	2.687
C	-1.915	-0.522	2.362
C	-2.21	-1.245	1.054
C	-1.292	-0.705	-0.095
C	0.21	-0.683	0.299
C	-0.206	-0.068	4.069
Cl	-2.192	-3.062	1.342
C	-1.52	-1.498	-1.396
N	-0.89	-2.678	-1.606
C	0.467	-2.875	-1.075
C	0.753	-2.145	0.24
C	-1.19	-3.404	-2.834
N	-2.353	-0.949	-2.239
C	-2.648	0.321	-1.721
C	-1.967	0.603	-0.515
C	-2.058	1.853	0.081
C	-2.86	2.819	-0.541
C	-3.559	2.546	-1.716
C	-3.455	1.288	-2.318
Cl	-2.981	4.419	0.187
H	-0.367	1.004	4.25
H	0.837	-0.29	4.288
H	-0.86	-0.642	4.73
H	-0.64	-2.996	-3.693
H	-2.256	-3.329	-3.045
H	-0.912	-4.453	-2.7
H	0.517	1.216	-0.696
H	0.961	-0.16	-1.686
H	-2.42	-1.044	3.177
H	-2.363	0.481	2.302
H	0.616	-3.949	-0.923
H	1.188	-2.564	-1.848
H	0.34	-2.712	1.073
H	1.839	-2.12	0.375
H	-3.247	-1.06	0.774
H	4.089	0.726	2.744
H	3.335	0.531	-2.157
H	6.012	1.012	1.171
H	-1.517	2.108	0.986
H	-4.174	3.319	-2.165
H	-3.978	1.068	-3.242

GIAO Chemical Shielding Tensors of the C26-inverted diastereomer of Caulamidine A

```
   1  C    Isotropic =    73.0726   Anisotropy =   141.7364
  XX=    60.9556   YX=   -16.8315   ZX=   -14.1683
  XY=   -20.4953   YY=   164.2705   ZY=    -3.9181
  XZ=   -10.5147   YZ=    -4.6094   ZZ=    -6.0084
  Eigenvalues:    -8.5422    60.1964   167.5635
   2  C    Isotropic =    55.9566   Anisotropy =   150.8524
  XX=     2.5270   YX=   -27.9479   ZX=    25.4012
  XY=   -14.4584   YY=   153.5974   ZY=     4.8041
  XZ=    35.5276   YZ=     5.7110   ZZ=    11.7454
  Eigenvalues:   -25.8132    37.1582   156.5249
   3  C    Isotropic =    73.5236   Anisotropy =   155.4043
  XX=    10.5344   YX=   -22.8742   ZX=   -21.5142
  XY=   -27.0330   YY=   173.3245   ZY=     3.8577
  XZ=   -14.5661   YZ=    -3.2979   ZZ=    36.7119
  Eigenvalues:    -1.5053    44.9497   177.1265
   4  C    Isotropic =    71.9046   Anisotropy =   124.9891
  XX=    71.0436   YX=   -12.2261   ZX=   -10.2891
  XY=    -5.1612   YY=   154.2610   ZY=    -5.0282
  XZ=   -15.2088   YZ=    -4.4484   ZZ=    -9.5907
  Eigenvalues:   -11.7769    72.2600   155.2307
   5  C    Isotropic =    62.4500   Anisotropy =    96.5695
  XX=    23.6753   YX=   -15.3945   ZX=    44.9970
  XY=   -16.1215   YY=   124.4183   ZY=     8.9655
  XZ=    44.6038   YZ=     5.7917   ZZ=    39.2565
  Eigenvalues:   -16.0218    76.5421   126.8297
   6  C    Isotropic =    71.2329   Anisotropy =   141.2294
  XX=     0.6765   YX=   -26.9316   ZX=   -27.0433
```

XY= -27.8733 YY= 160.8251 ZY= -3.7914
XZ= -24.2809 YZ= -5.6329 ZZ= 52.1970
Eigenvalues: -14.1415 62.4543 165.3858
7 Cl Isotropic = 745.2450 Anisotropy = 446.2478
XX= 853.8384 YX= 35.6811 ZX= -221.4033
XY= 37.7094 YY= 611.8026 ZY= -30.2275
XZ= -221.5818 YZ= -31.8769 ZZ= 770.0941
Eigenvalues: 586.5384 606.4532 1042.7435
8 N Isotropic = 26.1567 Anisotropy = 335.8053
XX= -98.8232 YX= -95.4050 ZX= 87.3591
XY= -126.4314 YY= 204.6895 ZY= -24.8479
XZ= 78.1603 YZ= -24.4276 ZZ= -27.3963
Eigenvalues: -171.5198 -0.0371 250.0268
9 C Isotropic = 40.6238 Anisotropy = 125.4938
XX= 33.3926 YX= -23.6023 ZX= 50.1005
XY= -43.0142 YY= 107.1832 ZY= -3.9342
XZ= 9.7521 YZ= -25.2015 ZZ= -18.7045
Eigenvalues: -32.3334 29.9184 124.2863
10 C Isotropic = 162.8777 Anisotropy = 30.0302
XX= 181.8235 YX= 8.7794 ZX= 4.2004
XY= 2.1444 YY= 153.8293 ZY= -8.7429
XZ= -3.5261 YZ= -7.1401 ZZ= 152.9804
Eigenvalues: 145.0066 160.7287 182.8979
11 N Isotropic = 175.9394 Anisotropy = 67.1626
XX= 147.0520 YX= -18.5302 ZX= -53.5811
XY= -28.2229 YY= 186.6066 ZY= -14.8983
XZ= -34.5243 YZ= -8.3281 ZZ= 194.1595
Eigenvalues: 111.6175 195.4862 220.7144
12 C Isotropic = 140.2180 Anisotropy = 28.4338
XX= 150.0455 YX= 13.1354 ZX= 9.8243
XY= 4.6848 YY= 129.4941 ZY= 3.4935
XZ= 7.2466 YZ= 8.9280 ZZ= 141.1145
Eigenvalues: 125.4945 135.9857 159.1739
13 C Isotropic = 128.8183 Anisotropy = 49.9079
XX= 95.8642 YX= -1.8113 ZX= -16.3194
XY= -15.9384 YY= 157.9407 ZY= -6.3444
XZ= -8.7745 YZ= -14.9197 ZZ= 132.6501
Eigenvalues: 89.9710 134.3938 162.0903
14 C Isotropic = 133.7180 Anisotropy = 18.6175
XX= 123.7419 YX= 2.5494 ZX= -4.2646
XY= -3.6369 YY= 145.9213 ZY= -2.9610
XZ= 8.2795 YZ= -0.3013 ZZ= 131.4907
Eigenvalues: 123.2518 131.7724 146.1296
15 C Isotropic = 154.1476 Anisotropy = 12.1008
XX= 150.1310 YX= -7.6120 ZX= -1.6769
XY= -0.1831 YY= 160.1872 ZY= 4.0248
XZ= 3.0396 YZ= 1.9806 ZZ= 152.1246
Eigenvalues: 148.0615 152.1664 162.2148
16 C Isotropic = 157.7108 Anisotropy = 50.8514
XX= 140.0679 YX= 1.4964 ZX= 8.0717
XY= 6.6306 YY= 145.0484 ZY= 6.0360
XZ= 14.1620 YZ= 7.0967 ZZ= 188.0161
Eigenvalues: 136.7266 144.7941 191.6117
17 Cl Isotropic = 766.7292 Anisotropy = 412.8943
XX= 598.9980 YX= 42.4141 ZX= -5.9603
XY= 77.1979 YY= 1014.7092 ZY= -59.8360
XZ= -65.5253 YZ= -95.1166 ZZ= 686.4805
Eigenvalues: 583.6721 674.5235 1041.9921
18 C Isotropic = 25.4558 Anisotropy = 122.5169
XX= 47.4305 YX= 54.3451 ZX= -24.0093
XY= 29.2342 YY= 9.8549 ZY= -71.3698
XZ= -18.8449 YZ= -46.5102 ZZ= 19.0820
Eigenvalues: -47.9317 17.1654 107.1338
19 N Isotropic = 167.5346 Anisotropy = 63.2250
XX= 155.6050 YX= -4.6320 ZX= -54.2999
XY= 15.2596 YY= 192.8483 ZY= 13.2262
XZ= -54.1783 YZ= 6.0392 ZZ= 154.1506
Eigenvalues: 99.4325 193.4867 209.6847
20 C Isotropic = 147.0466 Anisotropy = 50.3478
XX= 171.7934 YX= -8.0007 ZX= 20.4559
XY= -6.0492 YY= 128.8272 ZY= 7.6769
XZ= 16.6398 YZ= 7.7454 ZZ= 140.5192
Eigenvalues: 120.1968 140.3312 180.6118
21 C Isotropic = 168.9768 Anisotropy = 14.1585

XX= 156.8056 YX= -11.6043 ZX= 6.7550
XY= 3.1556 YY= 175.5379 ZY= 0.3226
XZ= 0.6458 YZ= 6.2455 ZZ= 174.5869
Eigenvalues: 154.9390 173.5756 178.4158
22 C Isotropic = 157.7931 Anisotropy = 53.8240
XX= 142.5332 YX= 7.0439 ZX= 8.2034
XY= 11.8922 YY= 155.7796 ZY= 22.9205
XZ= 12.8729 YZ= 21.9268 ZZ= 175.0664
Eigenvalues: 137.5097 142.1937 193.6758
23 N Isotropic = 19.2767 Anisotropy = 296.9113
XX= 106.4120 YX= 141.4694 ZX= -44.3249
XY= 147.8775 YY= -99.9986 ZY= -52.7645
XZ= -85.9960 YZ= -32.8864 ZZ= 51.4169
Eigenvalues: -174.8301 15.4427 217.2176
24 C Isotropic = 43.6027 Anisotropy = 131.9071
XX= 98.9899 YX= 21.9372 ZX= -33.8395
XY= 37.8348 YY= -6.8267 ZY= -36.4741
XZ= -39.6887 YZ= -46.9968 ZZ= 38.6448
Eigenvalues: -32.0606 31.3278 131.5407
25 C Isotropic = 61.6332 Anisotropy = 137.3777
XX= 91.2892 YX= 39.9991 ZX= -73.7072
XY= 36.7759 YY= 23.3759 ZY= -13.1472
XZ= -50.6631 YZ= -3.1069 ZZ= 70.2345
Eigenvalues: -2.1677 33.8490 153.2184
26 C Isotropic = 75.1787 Anisotropy = 133.1197
XX= 111.2542 YX= 11.6139 ZX= -68.5236
XY= 15.9729 YY= 67.8689 ZY= -28.0302
XZ= -71.2402 YZ= -31.1226 ZZ= 46.4129
Eigenvalues: -2.8504 64.4613 163.9252
27 C Isotropic = 65.0497 Anisotropy = 92.6541
XX= 109.0294 YX= 21.1990 ZX= -14.5893
XY= 17.6468 YY= 8.6163 ZY= -40.4671
XZ= -16.5828 YZ= -43.1243 ZZ= 77.5034
Eigenvalues: -12.1220 80.4519 126.8191
28 C Isotropic = 68.8994 Anisotropy = 145.7432
XX= 99.4149 YX= 56.8785 ZX= -64.5235
XY= 55.3271 YY= 31.7291 ZY= 2.4198
XZ= -63.2576 YZ= 4.1782 ZZ= 75.5542
Eigenvalues: -16.2686 56.9053 166.0615
29 C Isotropic = 79.0484 Anisotropy = 145.7763
XX= 119.3513 YX= 24.1829 ZX= -69.6280
XY= 22.9727 YY= 68.9400 ZY= -31.2888
XZ= -73.8502 YZ= -29.2136 ZZ= 48.8540
Eigenvalues: 1.2488 59.6639 176.2326
30 Cl Isotropic = 746.2618 Anisotropy = 449.2430
XX= 608.4787 YX= -26.8547 ZX= -5.6684
XY= -23.9231 YY= 966.0381 ZY= 174.6832
XZ= -20.8280 YZ= 169.1370 ZZ= 664.2687
Eigenvalues: 586.2946 606.7337 1045.7572
31 H Isotropic = 28.9001 Anisotropy = 11.0896
XX= 24.2237 YX= -0.2555 ZX= 0.3893
XY= -0.6789 YY= 31.5101 ZY= 4.7668
XZ= 0.6749 YZ= 5.3281 ZZ= 30.9665
Eigenvalues: 23.9949 26.4123 36.2932
32 H Isotropic = 27.3619 Anisotropy = 9.9109
XX= 29.9651 YX= -0.4537 ZX= 2.6419
XY= 0.1138 YY= 21.2501 ZY= -0.7230
XZ= 4.3674 YZ= -0.2396 ZZ= 30.8704
Eigenvalues: 21.2261 26.8904 33.9692
33 H Isotropic = 29.6070 Anisotropy = 10.3938
XX= 26.1952 YX= 1.8074 ZX= -0.7443
XY= 2.2310 YY= 26.7004 ZY= -1.3914
XZ= -1.9545 YZ= -2.1331 ZZ= 35.9255
Eigenvalues: 24.4106 27.8743 36.5362
34 H Isotropic = 29.0500 Anisotropy = 10.7675
XX= 25.4172 YX= 0.3726 ZX= -1.7401
XY= 0.9949 YY= 26.0561 ZY= 1.7197
XZ= -2.2989 YZ= 1.1940 ZZ= 35.6767
Eigenvalues: 24.4319 26.4898 36.2284
35 H Isotropic = 27.8186 Anisotropy = 10.4796
XX= 29.8547 YX= 0.7275 ZX= 4.1666
XY= 2.0011 YY= 26.1105 ZY= 4.1969
XZ= 5.1044 YZ= 3.1951 ZZ= 27.4907
Eigenvalues: 22.3285 26.3223 34.8050

36 H Isotropic = 29.4073 Anisotropy = 10.6345
XX= 24.8652 YX= 0.4888 ZX= 1.3427
XY= -0.1989 YY= 34.6916 ZY= 3.0466
XZ= 0.8446 YZ= 4.3933 ZZ= 28.6650
Eigenvalues: 24.4803 27.2446 36.4969
37 H Isotropic = 29.0263 Anisotropy = 10.0074
XX= 34.6552 YX= -1.9856 ZX= 1.6370
XY= -2.6608 YY= 30.0600 ZY= -2.9541
XZ= -1.5409 YZ= -1.7945 ZZ= 22.3637
Eigenvalues: 21.6624 29.7186 35.6979
38 H Isotropic = 29.8642 Anisotropy = 5.9386
XX= 31.5221 YX= 1.9642 ZX= -0.1533
XY= 3.9720 YY= 25.6185 ZY= 0.5313
XZ= -2.8476 YZ= 0.7169 ZZ= 32.4520
Eigenvalues: 24.2199 31.5495 33.8232
39 H Isotropic = 28.4720 Anisotropy = 7.5985
XX= 28.3086 YX= 3.2901 ZX= -4.1830
XY= 2.1209 YY= 25.5675 ZY= 0.5455
XZ= -1.9337 YZ= 0.2750 ZZ= 31.5398
Eigenvalues: 23.3832 28.4950 33.5377
40 H Isotropic = 27.6224 Anisotropy = 6.8133
XX= 25.9434 YX= -3.8591 ZX= -1.7468
XY= -4.9846 YY= 28.9917 ZY= 0.0283
XZ= 1.2949 YZ= 0.3636 ZZ= 27.9323
Eigenvalues: 22.7894 27.9133 32.1647
41 H Isotropic = 28.7127 Anisotropy = 8.7067
XX= 26.7708 YX= -4.2686 ZX= 1.3686
XY= -1.6291 YY= 33.2221 ZY= 2.1163
XZ= 1.4970 YZ= 1.3396 ZZ= 26.1452
Eigenvalues: 23.8574 27.7636 34.5172
42 H Isotropic = 28.6337 Anisotropy = 7.7090
XX= 32.1196 YX= 0.0499 ZX= -3.4035
XY= 1.8174 YY= 25.2512 ZY= 3.7017
XZ= -2.3200 YZ= 2.5739 ZZ= 28.5303
Eigenvalues: 22.7627 29.3654 33.7731
43 H Isotropic = 28.7987 Anisotropy = 6.3427
XX= 27.5215 YX= -0.5579 ZX= -1.3805
XY= 2.3700 YY= 29.5225 ZY= -2.7021
XZ= -3.2270 YZ= -2.5719 ZZ= 29.3521
Eigenvalues: 25.7343 27.6347 33.0272
44 H Isotropic = 30.0998 Anisotropy = 7.6128
XX= 34.3046 YX= -0.5201 ZX= 2.3734
XY= 2.3838 YY= 30.8911 ZY= 1.9042
XZ= 2.1477 YZ= 0.6851 ZZ= 25.1036
Eigenvalues: 24.4045 30.7198 35.1750
45 H Isotropic = 27.9646 Anisotropy = 6.3038
XX= 29.5260 YX= 0.9456 ZX= -1.4573
XY= -3.2224 YY= 29.7933 ZY= -4.2581
XZ= 1.4553 YZ= -3.3055 ZZ= 24.5746
Eigenvalues: 22.5484 29.1783 32.1672
46 H Isotropic = 24.4928 Anisotropy = 6.5158
XX= 28.5241 YX= 1.7164 ZX= 0.0133
XY= 1.4864 YY= 20.6077 ZY= 0.3579
XZ= -0.3000 YZ= 0.4524 ZZ= 24.3466
Eigenvalues: 20.2519 24.3898 28.8367
47 H Isotropic = 24.8762 Anisotropy = 11.1019
XX= 31.4931 YX= 2.2233 ZX= -1.2732
XY= 2.5321 YY= 20.0388 ZY= -0.1269
XZ= -2.0670 YZ= -0.3873 ZZ= 23.0968
Eigenvalues: 19.5631 22.7880 32.2775
48 H Isotropic = 24.4658 Anisotropy = 5.9668
XX= 25.0480 YX= 0.9693 ZX= -1.0386
XY= 0.9883 YY= 20.0704 ZY= 0.1332
XZ= -0.4561 YZ= 0.2131 ZZ= 28.2789
Eigenvalues: 19.8733 25.0804 28.4436
49 H Isotropic = 24.8185 Anisotropy = 12.6255
XX= 22.1256 YX= -4.5441 ZX= 1.7907
XY= -5.8327 YY= 30.5622 ZY= -0.9342
XZ= 1.5236 YZ= -0.8695 ZZ= 21.7677
Eigenvalues: 19.1542 22.0658 33.2355
50 H Isotropic = 24.3909 Anisotropy = 6.0922
XX= 22.2968 YX= -0.7976 ZX= 3.1676
XY= -0.7974 YY= 24.9888 ZY= 2.3042
XZ= 3.2676 YZ= 1.9753 ZZ= 25.8871

Eigenvalues: 19.7744 24.9460 28.4524
51 H Isotropic = 24.3909 Anisotropy = 4.8787
XX= 22.9875 YX= -2.1806 ZX= 2.6375
XY= -2.5389 YY= 26.0972 ZY= 0.2203
XZ= 2.2698 YZ= 0.4870 ZZ= 24.0878
Eigenvalues: 20.2613 25.2680 27.6433

References

1. C. Robinson. *Trans. Faraday Soc.* 1956, **52**, 571.
2. C. Robinson, J.C. Ward and R.B. Beevers. Diss. Faraday Soc. 1958, 25, 29.
3. C. Robinson. *Tetrahedron* 1961, **13**, 219.
4. I. Canet, A. Meddour, J. Courtieu. *J. Am. Chem. Soc.* 1994, **116**, 2155.
5. G. Bifulco, R. Riccio, G. E. Martin, A. V. Buevich, R. T. Williamson. *Org. Lett.* 2013, **15**, 654.
6. B. P. Feuston, M. D. Miller, J. C. Culberson, R. B. Nachbar, S. K. Kearsley. *J. Chem. Inf. Comput. Sci.* 2001, **41**, 754.
7. J. M. Blaney, G. M. Crippen, A. Dearing, J. S. Dixon. DGEOM; Quantum Chemistry Program Exchange; Indiana University, Bloomington, 1990.
8. a) P. C. Hawkins, A. Nicholls. *J. Chem. Inf. Model.* 2012, 52, 2919-2936; b) P. C. Hawkins, A. G. Skillman, G. L. Warren, B. A. Ellingson, M. T. Stahl. *J. Chem. Inf. Model.* 2010, **50**, 572.
9. M. J. Frisch, G. W. Trucks, H. B. Schlegel, G. E. Scuseria, M. A. Robb, J. R. Cheeseman, G. Scalmani, V. Barone, G. A. Petersson, H. Nakatsuji, X. Li, M. Caricato, A. Marenich, J. Bloino, B. G. Janesko, R. Gomperts, B. Mennucci, H. P. Hratchian, J. V. Ortiz, A. F. Izmaylov, J. L. Sonnenberg, D. Williams-Young, F. Ding, F. Lipparini, F. Egidi, J. Goings, B. Peng, A. Petrone, T. Henderson, D. Ranasinghe, V. G. Zakrzewski, J. Gao, N. Rega, G. Zheng, W. Liang, M. Hada, M. Ehara, K. Toyota, R. Fukuda, J. Hasegawa, M. Ishida, T. Nakajima, Y. Honda, O. Kitao, H. Nakai, T. Vreven, K. Throssell, J. A. Montgomery, Jr., J. E. Peralta, F. Ogliaro, M. Bearpark, J. J. Heyd, E. Brothers, K. N. Kudin, V. N. Staroverov, T. Keith, R. Kobayashi, J. Normand, K. Raghavachari, A. Rendell, J. C. Burant, S. S. Iyengar, J. Tomasi, M. Cossi, J. M. Millam, M. Klene, C. Adamo, R. Cammi, J. W. Ochterski, R. L. Martin, K. Morokuma, O. Farkas, J. B. Foresman, and D. J. Fox, Gaussian, Inc., Wallingford CT, 2016.
